# A small-molecule compound D6 overcomes EGFR-T790M-mediated resistance in non-small cell lung cancer

**DOI:** 10.1038/s42003-021-02906-4

**Published:** 2021-12-13

**Authors:** Xiaolong Tang, Lizhi Cheng, Guo Li, Yong-Ming Yan, Fengting Su, Dan-Ling Huang, Shuping Zhang, Zuojun Liu, Minxian Qian, Ji Li, Yong-Xian Cheng, Baohua Liu

**Affiliations:** 1 Shenzhen Key Laboratory for Systemic Aging and Intervention (SKL-SAI), School of Basic Medical Sciences; Institute for Inheritance-Based Innovation of Chinese Medicine, School of Pharmaceutical Sciences, Shenzhen University, Shenzhen, China; 2 Department of Dermatology, Xiangya Hospital, Central South University, Changsha, China; 3 Guangdong Key Laboratory of Genome Stability and Human Disease Prevention, Shenzhen University, Shenzhen, China; 4 National Engineering Research Center for Biotechnology (Shenzhen); Marshall Laboratory of Biomedical Engineering; International Cancer Center, Shenzhen University, Shenzhen, China; 5 Shenzhen Bay Laboratory, Shenzhen, China

**Keywords:** Non-small-cell lung cancer, Target validation

## Abstract

Non-small cell lung cancer (NSCLC) is a deadly and highly prevalent malignancy. Targeting activated-EGFR mutations in NSCLC via EGFR tyrosine kinase inhibitor (EGFR-TKI) initially achieves a profound therapeutic response, but resistance frequently evolves, reducing treatment options. Here, we present a small-molecule compound D6 which selectively inhibits tumor cell growth and migration in NSCLC cells with EGFR-TKI-resistant T790M-EGFR-activated mutations (T790M-EGFR-AM), e.g., L858R/T790M, 19Del/T790M and L858R/T790M/C797S. D6 mimics a natural product isolated from the roots of *Codonopsis pilosula* and selectively competes with T790M-EGFR-AM to bind to HSP90, thus facilitating the ubiquitination dependent proteasomal degradation of T790M-EGFR-AM. By contrast, D6 has little impact on typical HSP90 chaperone activity, suggesting low systemic toxicity. Promisingly, D6 combined with erlotinib or osimertinib shows efficacy in overcoming the EGFR-TKIs-resistance in NSCLCs. Our study raises an alternative strategy to overcome T790M-mediated EGFR-TKI resistance in NSCLC via targeting the protein–protein interaction of HSP90 and T790M-EGFR by intervention with D6.

## Abstract (web summary)

Tang et al. propose the use of a small molecule compound D6 to treat EGFRTKI resistant tumors. The authors demonstrate potent and selective inhibition of NSCLC with T790M-mediated EGFR-TKI resistance through inhibiting HSP90-mediated protein–protein interaction.

## Introduction

Lung cancer is the worldwide leading cause of cancer-related deaths^1^. Non-small cell lung cancer (NSCLC) constitutes almost 90% of lung cancer patients and has a poor prognosis with a 5-year survival of ∼15%^2,3^. Epidermal growth factor receptor (EGFR), also known as class I receptor tyrosine kinase, utilizes the tyrosine kinase (TK) domain to transduce extracellular signals upon ligand binding, e.g., EGF^4^. Activated EGFR signaling plays critical roles in driving NSCLC tumorigenesis and progression by activating two major downstream pathways in a kinase-dependent manner, i.e., the Ras/ERK1/2 and the PI3K/AKT^4,5^. EGFR typically exists in an autoinhibited conformation, and somatic mutations which destabilize this conformation can result in a ligand-independent activation: these mutations are widely observed and account for about 20% of advanced NSCLCs^6^. Most EGFR-activated mutations display as in-frame deletions in exon 19 E746_A750 (19Del-EGFR) or an L858R point mutation (L858R-EGFR)^6–8^.

NSCLCs harboring EGFR-activated mutations are more sensitive to EGFR tyrosine kinase inhibitors (EGFR-TKIs). The first generation of TKIs (erlotinib and gefitinib) are widely applied for 19Del- or L858R-EGFR-positive patients^9^. While initially achieving tumor regression, most patients develop acquired resistance 9–13 months post therapy^10,11^. Approximately 60% acquired resistance is attributed to an additional T790M mutation—the methionine substitution generates a bulky amino acid side chain which conducts a steric hindrance and thus interferes the binding of gefitinib or erlotinib to the EGFR^12–14^. To overcome the T790M-EGFR resistance, osimertinib, a representative third generation of EGFR-TKIs, which acts irreversibly and selectively for EGFR-activating mutations including T790M mutation, has been widely applied in clinic^15,16^. However, after approximately 10 months of exposure, tumor cells develop resistance, e.g., the C797 and L792 residues of T790M-EGFR are mutated with a portion of ∼26%^15,17–19^. Exploring new strategies to overcome the continuous acquisition of EGFR-TKI resistance is thus clinically urgent.

One avenue being pursued to overcome EGFR-TKI resistance is HSP90 inhibition. HSP90 belongs to the heat-shock protein family and assists protein folding or refolding^20^ and oncogenic proteins levels, maturation, and continued dysregulated activity are regulated by HSP90 chaperone activity in many cancers^21^. TKI-resistant T790M-EGFR NSCLC are more sensitive than wild-type (WT) cells in response to an HSP90 inhibitor which promotes fast degradation of T790M-EGFR to prevent tumor progression^22–24^. Thus far, most HSP90 inhibitors are designed to overcome the EGFR-TKI resistance by targeting either N- or C-terminus^25^, e.g., 17-AAG (tanespimycin), 17-DMAG (alvespimycin) and IPI-504 (retaspimycin hydrochloride), which elicit anti-neoplastic activity in NSCLC^26–28^. However, at present there are few approved HSP90 inhibitors because the severe toxicity is observed from clinic trials, such as induction of heat-shock response, retinopathy and gastrointestinal tract toxicity^29^. Development of HSP90 inhibitors with acceptable toxicity may provide an alternative strategy to overcome the EGFR-TKI resistance in NSCLCs.

The root of *Codonopsis pilosula* (*Dangshen* in Chinese) is a traditional Chinese medicine which is usually applied as an adjuvant to improve immune function and ameliorate therapy-induced fatigue for cancer patients^30,31^. Compounds isolated from *C. pilosula* have potentials to inhibit cancer cell growth or migration^32,33^. Here, we identified a small-molecule compound **D6**, a synthetic derivative of natural compound from *C. pilosula*, which possesses selective activity on NSCLC cells harboring T790M-EGFR mutation by targeting HSP90. In combination with erlotinib or osimertinib, D6 has potential to prevent the acquired EGFR-TKI resistance in NSCLC.

## Results

### Chemical structure of D6

We initially obtained a group of natural small molecules from the root of *C. pilosula* (n-D5, n-D6 and n-D7) and tested their anti-tumor activity in NSCLC cells. Interestingly, n-D6 (methyl (*Z*)-4-oxo-4-(9*H*-pyrido [3, 4-b] indol-1-yl) but-2-enoate) (hereafter code: **D6-1**, Supplementary Figs. 1–6) had promising anti-tumor activity. For further large-scale biological analysis, an analogue of **D6-1**, i.e., **D6**, was synthesized by a natural product derivative tryptamine as described in Supplementary methods (Scheme 1) and Supplementary Fig. 7. As shown, **D6** is an HPLC-grade pure small-molecular compound (Supplementary Fig. 8). Its planar structure was further identified by spectroscopic methods including ^1^H, ^13^C NMR, ^1^H-^1^H COSY, HSQC, HMBC, and HRESIMS spectrum (Supplementary Figs. 9–17). The geometry of double bond was assigned as *cis* form by the coupling *J* = 12.2 Hz. Interestingly, the *cis*-**D6** almost completely transformed to *trans* form in the cell culture medium (Supplementary Figs. 18 and 19), which indicates that the effect of **D6** in our following performance was indeed elicited by the *trans*-**D6**.

### NSCLC cells expressing L858/T790M-EGFR are sensitive to D6

**Fig. 1:**
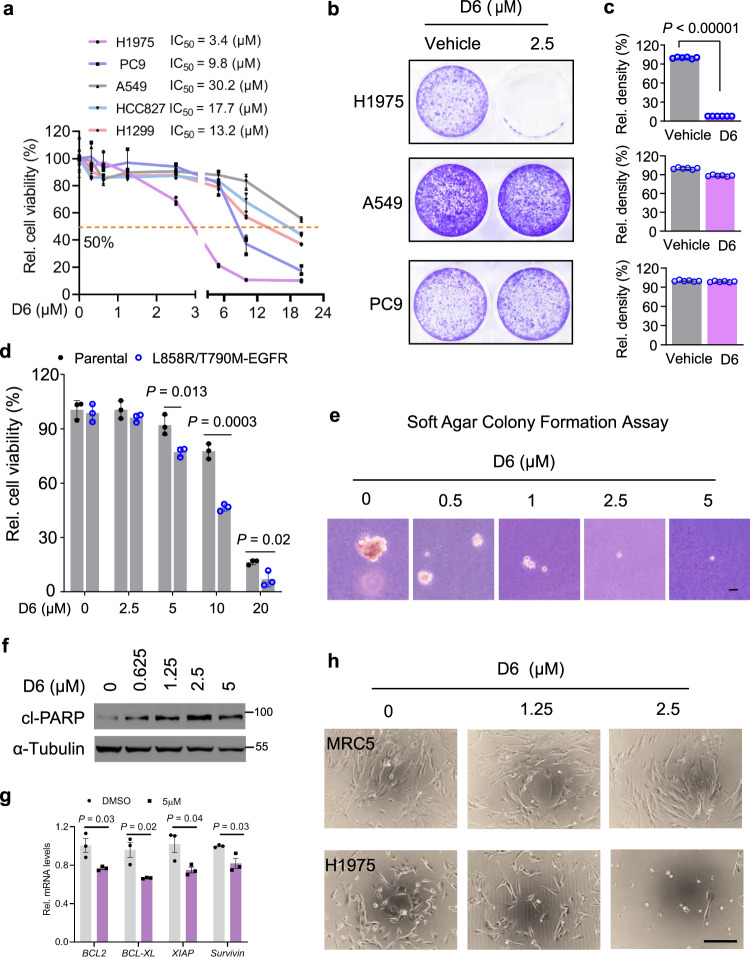
D6 inhibits the viability of NCI-H1975 cells. **a** PC9, H1299, A549, HCC827, and NCI-H1975 cells were treated with a range of concentrations of **D6** for 72 h. Cell viability was detected by CCK8 and shown as relative viability compared to the control. **b**, **c** Representative images showed the cell proliferation of NCI-H1975, A549, and PC9 cells exposed to **D6** (2.5 µM) and solvent DMSO. The bars of (**c**) represented the relative cell density. **d** HCC827 parental cells or the isogenic cells expressing L858R/T790M-EGFR were treated with the indicated concentrations of **D6** for 72 h. Cell viability (%) was shown after normalized to the control group. **e** Representative images showed the cell growth of NCI-H1975 cells cultured in soft agar with the indicated concentrations of **D6**. Scale bar, 200 µm. **f** Immunoblotting analysis showing the levels of cleaved-PARP (cl-PARP) in NCI-H1975 cells treated with indicated doses of **D6**. **g** Quantitative PCR analysis showing the levels of anti-apoptosis genes in NCI-H1975 cells in the presence of **D6** or not. **h** Representative images showing the effect of **D6** on NCI-H1975 cells and normal lung fibroblast MRC5 cells. Scale bar, 200 µm. Data are presented as mean ± SEM (**a**, **c**, and **g**), *n* = 3 (**a** and **g**) or 6 (**c**) biologically independent samples. *P* values were calculated by two-tailed Student’s *t*-test.

We next asked whether **D6** had the similar anti-tumor activity as the natural product n-D6 for NSCLCs. Initially, three well-characterized human NSCLC cell lines (A549, PC9, and NCI-H1975) were treated with serial doses of **D6** for 72 h and then cell viability was detected by CCK-8 assay. **D6** reduced the cell viability of all NSCLC cells, showing IC_50_ values of 30.2 μM for A459, 9.8 μM for PC9, and 3.4 μM for NCI-H1975 (Fig. 1a). Notably, NCI-H1975 cells were more sensitive to **D6**.

One factor that may explain the differing responses observed between NSCLC cells is their EGFR status. Verified by genomic DNA sequencing, as shown, A549 cells have WT EGFR; PC9 cells express 19 Del (E746_A750) EGFR (19Del-EGFR); and NCI-H1975 cells possess L858R/T790M-EGFR^3^ (Supplementary Fig. 20a). Consistent with the intrinsic EGFR status, PC9 cells were most sensitive to erlotinib treatment, while A549 and NCI-H1975 cells showed apparent non-response or resistance (Supplementary Fig. 20b). Since NCI-H1975 cells expressing EGF-TKIs-resistant L858R/T790M-EGFR were more sensitive to **D6**, we investigated if **D6** selectively targets the L858R/T790M-EGFR mutated NSCLCs. To reduce the impact of cell-context-dependent effects, we employed another two human NSCLC cell lines, HCC827 and H1299, which express 19Del-EGFR and WT EGFR, respectively. Both HCC827 and H1299 cells displayed much higher IC_50_ does of **D6** than NCI-H1975 cells (Fig. 1a). Consistently, cell proliferation assays confirmed that **D6** at the dose of 2.5 μM achieved marked cell growth arrest for NCI-H1975 cells but had marginal effect on either A549 or PC9 cells (Fig. 1b, c). To test the possibility that **D6** preferentially inhibits L858/T790M-EGFR addicted NSCLC cells, we generated an isogenic cell line from HCC827 by stably expressing L858R/T790M-EGFR and then selected by erlotinib, to mimic the erlotinib resistance. **D6** consistently displayed increased capacity to kill L858R/T790M-EGFR-expressing cells compared to their parental cells (Fig. 1d), indicating that the effect was not cell-type dependent.

Given that targeting the T790M-EGFR NSCLCs has clinical relevance, further analyses were performed in NCI-H1975 cells. **D6** treatment at the dose 0.5 μM (lower than IC_50_ (3.4 μM)) was sufficient to suppress growth of NCI-H1975 cells in soft agar, which mimics tumor progress in vivo^34^ (Fig. 1e). Moreover, 48 h post exposure to **D6**, we observed a dose-dependent increase of cleaved PARP, a hallmark of apoptosis^35^, in NCI-H1975 cells, suggesting that **D6** treatment eventually elicits cell apoptosis (Fig. 1f). Similarly, a significant downregulation of anti-apoptotic genes was observed after **D6** treatment (Fig. 1g). Importantly, noncancerous cells, such as MRC5 (normal lung fibroblasts), HEK293, and LO2, were less sensitive to **D6** in comparison with NCI-H1975 cells, suggesting a lower toxicity (Fig. 1h and Supplementary Fig. 20c). Together, these results suggest that **D6** potently targets the NSCLC cells harboring L858R/T790M-EGFR.

### D6 inhibits NCI-H1975 cell migration and invasion

**Fig. 2:**
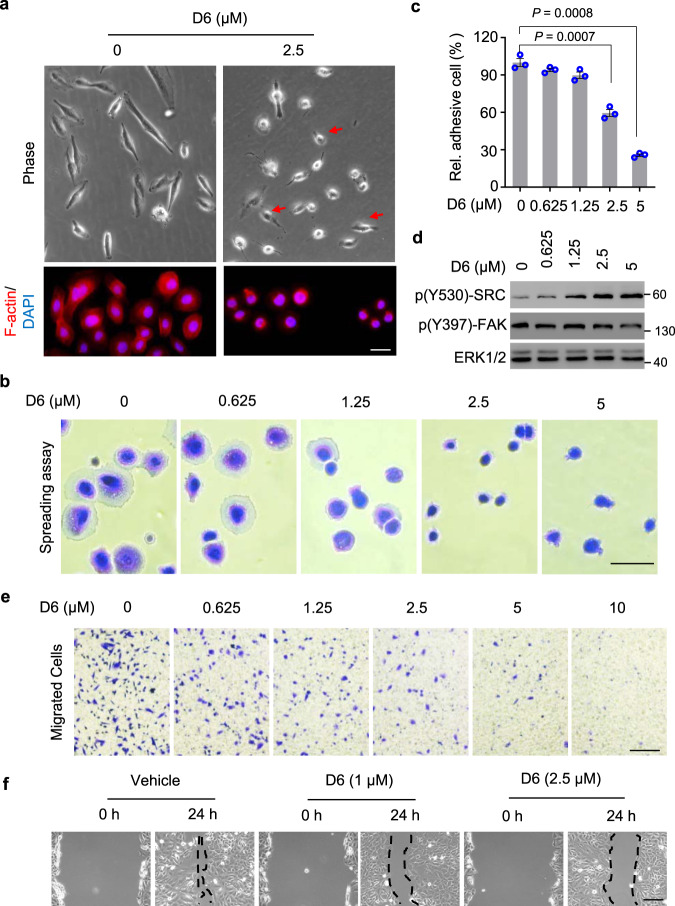
D6 inhibits NCI-H1975 cell migration. **a** Representative images showing the cell morphology of NCI-H1975 cells post **D6** exposure. Scale bar, 100 µm. **b**, **c** Representative images (**b**) showing NCI-H1975 cells spreading on cell culture plate with **D6** incubation or not. The adhesive cells were counted and normalized to the solvent control (**c**). Scale bar, 50 µm. **d** Immunoblotting analysis of cell lysates prepared from NCI-H1975 cells exposed to different concentrations of **D6**. **e** NCI-H1975 cells with **D6** pretreatment were subjected to transwell assay. The migrated cells were stained by 0.1% crystal violet solution. Scale bar, 200 µm. **f** Representative images showing the wound-healing assay for NCI-H1975 cells followed with **D6** treatments. The dash line indicated the edge of wound closure. Scale bar, 200 µm. Data represent mean ± SEM (**c**), *n* = 3 biologically independent samples. *P* values were calculated by two-tailed Student’s *t*-test.

Approximately 30–40% of NSCLC patients have metastatic tumors after diagnosis, which is the main cause of death^36^. Tumor metastasis is a complex process largely dependent on enhanced cell motility^37,38^. Interestingly, with **D6** treatment, NCI-H1975 cells shifted from spindle morphology to rounded morphology, which indicates that **D6** might contribute to regulation of cell adhesion and spreading (Fig. 2a). Assessed by a cell spreading assay, **D6** suppressed the adhesion of NCI-H1975 cells in a dose-dependent manner (Fig. 2b, c), noting that cell viability was marginally affected (Supplementary Fig. 21a). Cell adhesion and spreading are crucially regulated by SRC and FAK, two focal adhesion kinases^39^. Phosphorylation of SRC at Y530 (p-Y530) impairs its kinase activity and leads to downregulate (p-Y397) FAK, which disrupts the focal adhesion turnover and impairs cell migration^40,41^. **D6** treatment increased (p-Y530) SRC but decreased (p-Y397) FAK in the NCI-H1975 cells (Fig. 2d). Cell migration also relies on efficient focal adhesion turnover^42^. We examined the effect of **D6** on cell migration and showed that **D6** treatment suppressed NCI-H1975 cells evading through transwell in a dose-dependent manner (Fig. 2e). Similarly, **D6** treatment inhibited the migration of NCI-H1975 cells as determined by wound-healing assays (Fig. 2f). Of note, we did not observe apparent decrease of cell viability by **D6** treatment in those assays (Supplementary Fig. 21b, c). Together, the results indicate that **D6** has the potential to combat metastasis in NSCLCs.

### D6 decreases EGFR expression in NCI-H1975 cells

**Fig. 3:**
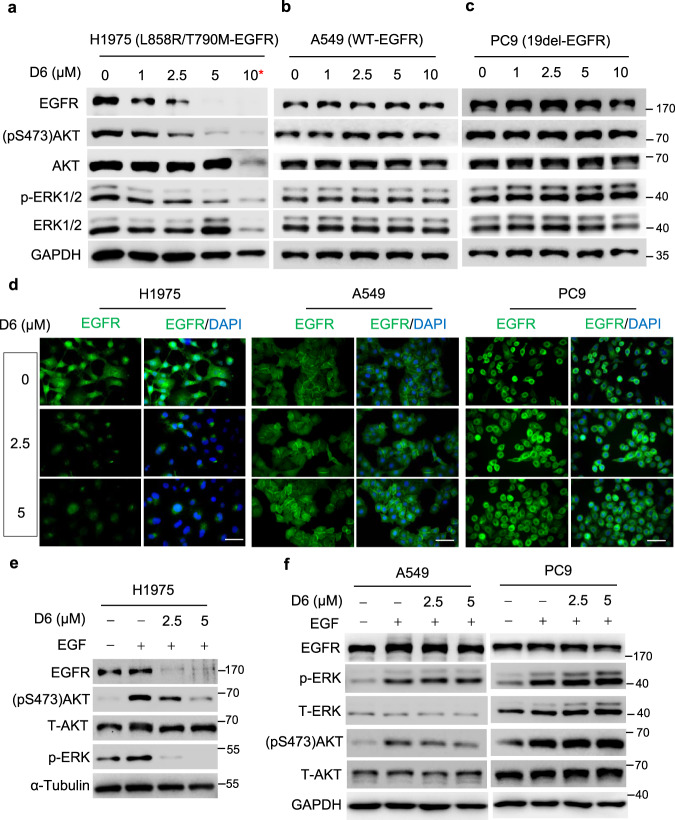
D6 suppressed EGFR expression in NCI-H1975 cells. **a−c** NCI-H1975, A549, and PC9 cells were treated with indicated concentrations of **D6** for 24 h. Cell lysates were prepared and subjected to immunoblotting analysis. *D6 resulted in cell death at the dose of 10 μM and led to total protein decrease. **d** Representative images showing the EGFR levels evaluated by immunofluorescent staining. NCI-H1975, A549, and PC9 cells were treated with **D6** for 24 h and then fixed, permeabilized and incubated with anti-EGFR antibody. After incubation with an FITC-conjugated secondary antibody, nuclei were stained with DAPI. Scale bars, 50 µm. **e**, **f** Immunoblotting analysis of cell lysates derived from NCI-H1975, A549, and PC9 cells with or without **D6** and EGF (10 ng/µl) treatment.

EGFR-mutant NSCLC cells are addicted to EGFR activity and require this activity to drive tumor growth, survival and metastasis^43^. **D6** treatment leads to a dose-dependent decline of EGFR protein expression in NCI-H1975 cells, concurring with the inhibition of EGFR downstream effectors like AKT and ERK1/2 (Fig. 3a). By contrast, **D6** treatment at the same dosages had a negligible effect on EGFR levels in both A549 and PC9 cells (Fig. 3b, c). Consistent results were observed by immunofluorescent staining of EGFR, where a prominent reduction of EGFR in NCI-H1975 cells but not in PC9 and A549 cells post **D6** treatment was observed (Fig. 3d). **D6** treatment markedly abrogated EGF-induced AKT and ERK activation in NCI-H1975 cells, but exerted no apparent effect on A549 and PC9 cells (Fig. 3e, f). These results indicate that **D6** treatment elicits selective anti-tumor efficacy in L858R/T790M-EGFR NSCLC cells by downregulating EGFR expression.

### D6 promotes T790M-EGFR degradation through the ubiquitin-proteasome system

**Fig. 4:**
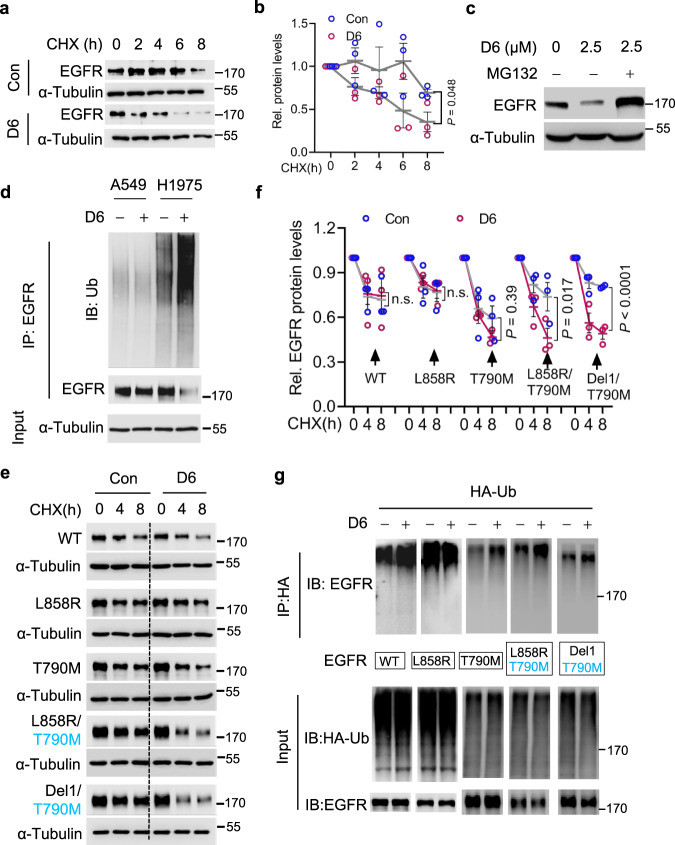
D6 promotes L858R/T790M-EGFR degradation. **a**, **b** Immunoblotting analysis (**a**) of cell lysates derived from NCI-H1975 cells treated by CHX (50 μg/ml) in the presence or absence of **D6** (2.5 μM); protein quantification is presented in (**b**), *n* = 3 independent experiments. *P* values were calculated by two-way ANOVA analysis. **c** EGFR protein levels were detected by immunoblotting in NCI-H1975 cells treated by **D6** (2.5 μM) with or without MG132 (10 μM). **d** NCI-H1975 or A549 cells transfected with HA-Ub were then treated with or without **D6** (2.5 μM) for 6 h. Cell lysates were immunoprecipitated with anti-HA beads and beads elution was further analyzed by immunoblotting. **e**, **f** Immunoblotting analysis (**e**) of cell lysates derived from HEK293 cells transfected with different EGFR mutants and then were subjected to CHX (50 μg/ml) chase assay with or without **D6** (2.5 μM) incubation. Protein degradation rate was shown as curves in (**f**), *n* = 3 independent experiments. *P* values were calculated by two-way ANOVA analysis. **g** Immunoblotting analysis of the elution of anti-HA beads prepared from HEK293 cells transfected with HA-Ub and EGFR mutant with or without **D6** (2.5 μM) exposure. Data are presented as mean ± SEM (**b** and **f**). Representative results were analyzed from at least three independent experiments.

While decreasing EGFR protein expression, **D6** treatment had little impact on the *EGFR* mRNA levels in NCI-H1975 cells (Supplementary Fig. 22), suggesting a potential effect on EGFR protein stability. We performed cycloheximide (CHX) chase assays, an experiment to measure protein stability^44^. D6 markedly facilitated EGFR protein turnover in NCI-H1975 cells (Fig. 4a, b) and this effect was potentially dependent on proteasome system as MG-132, a proteasome inhibitor^45^, nearly restored EGFR protein levels even with continued **D6** treatment (Fig. 4c). This finding is consistent with a previous report that the degradation of mutated EGFR protein is at least partially dependent on the proteasome^46^. Supporting such notion, **D6** treatment resulted in an obvious increase of ubiquitinated EGFR in NCI-H1975 cells. By contrast, **D6** treatment had little effect on A549 cells which express the WT EGFR (Fig. 4d).

To confirm that **D6** preferentially targets L858R/T790M-EGFR in NSCLC, we asked which architecture of EGFR is indispensable in response to **D6**, i.e., L858R, T790M alone or both; we also investigated if the observed effects are cell-type specific. We employed HEK293 cells which are entirely etiologically distinct to NCI-H1975 cells and performed CHX chase assay to evaluate the effect of **D6** on several regularly mutated EGFRs. **D6** treatment only marginally affected the stability of WT-EGFR, L858R-EGFR, and T790M-EGFR in HEK293 cells (Fig. 4e, f). However, L858R/T790M-EGFR and Del19/T790M-EGFR, two EGFR mutants that frequently emerge as TKI-resistant when patients are treated with gefitinib or erlotinib^10^, displayed prominent sensitivity to **D6** exposure (Fig. 4e, f). Consistently, **D6** increased the levels of ubiquitinated L858R/T790M-EGFR and Del19/T790M-EGFRs (Fig. 4g). These results suggest that **D6** preferably targets EGFR which simultaneously harbors kinase activation (L858R or Del19) and erlotinib-resistant (T790M) mutations.

### D6 compromises the interaction of T790M/L858R-EGFR and HSP90

**Fig. 5:**
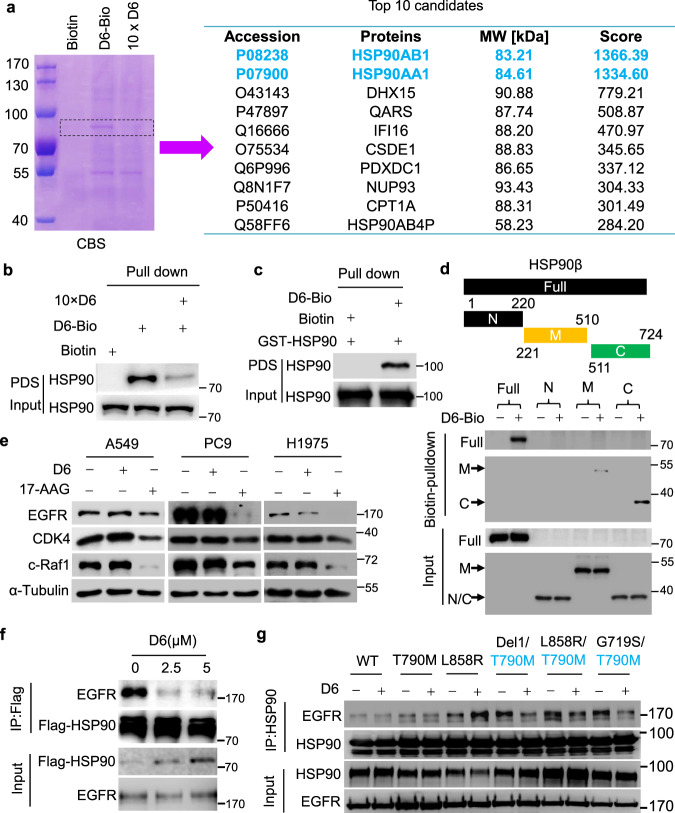
D6 attenuates the binding of T790M-EGFR mutants to HSP90. **a** Coomassie blue staining (CBS) showing proteins trapped by biotin-labeled **D6** (**D6-4**) from NCI-H1975 cell lysates. 10 × D6: using 10-fold **D6** to saturate the target proteins and thus excluding non-specific binding. The protein gel indicated by dashed line was further analyzed by protein mass spectrometry and identified proteins are listed in the right panel. **b**, **c** Immunoblotting analysis of the protein elution based on biotin–avidin pulldown assays from NCI-H1975 cell lysates (b) or purified recombinant GST-HSP90 protein (**c**); PDS: pulldown solution. **d** Immunoblotting analysis showing the domains where **D6** binds to HSP90. **e** Immunoblotting analysis of the cell lysates from A549, PC9, and NCI-H1975 cells treated with **D6** (2.5 μM) or 17-AAG (2.5 μM), respectively. **f** Immunoblotting analysis of the anti-HSP90 immunoprecipitates prepared from cell lysates of NCI-H1975 cells with indicated concentrations of **D6** treatment. **g** Immunoblotting analysis of anti-HSP90 immunoprecipitates derived from cell lysates of HEK293 cells transfected with HSP90 and indicated EGFR mutants with or without **D6** treatment.

To explore the mechanism that how **D6** impacts EGFR stability, we prepared biotin-labeled **D6**, i.e., **D6-4**, as shown in Supplementary methods (Scheme 2), and then performed biotin-based pulldown assay to identify **D6** targeting proteins. Interestingly, **D6-4** specifically captured some proteins with a molecular weight around ∼90 kDa (Fig. 5a, left), noting that 10 folded **D6** successfully competed such bindings. Via protein mass spectrometry, we identified HSP90 as a **D6** binding candidate (Fig. 5a, right). To confirm this, biotin-pulldown elution based on **D6-4** derived from NCI-H1975 cell lysates was applied for immunoblotting. HSP90 was apparently trapped by **D6-4**, while **D6** successfully antagonized the binding (Fig. 5b). The direct interaction of **D6** and HSP90 was verified through the pulldown assay of **D6-4** and purified HSP90 protein in vitro (Fig. 5c). HSP90 contains three major domains which rationally constitutes an ATP-dependent chaperone complex to regulate protein folding and degradation, i.e., the amino-terminal domain (NTD), the middle domain (MD) and the carboxy-terminal domain (CTD)^47^. To precisely define the **D6** binding region, we performed domain-truncation analysis. Both MD and CTD were detected in the elution based on **D6-4**, but the CTD domain had the strongest affinity to **D6** (Fig. 5d). These findings support that **D6** directly targets HSP90.

NSCLC cells harboring EGFR-activated mutations are much more sensitive to HSP90 inhibition than the wild type^23,24^. We then determined if **D6** is a HSP90 inhibitor. We observed that 17-AAG, an HSP90 inhibitor targeting the NTD of ATP binding pocket, obviously downregulated expression of general clients, like EGFR, CDK4, and c-Raf1, in human NSCLC PC9, NCI-H1975, and A549 cells (Fig. 5e). However, **D6** treatment had minor effect on these clients in all the tested cells with the exception of EGFR in NCI-H1975 (Fig. 5e), which suggests that **D6** is unlikely to be a typical HSP90 inhibitor. Additionally, whilst **D6** possessed a higher binding capacity to CTD (Fig. 5d) which is supposed to mediate HSP90 dimerization^48^, there was not a prominent decrease of HSP90 dimer formation in the presence of **D6** (Supplementary Fig. 23a). Recently, targeting HSP90 protein–protein interactions (PPIs) is considered as an alternative strategy to avoid the system toxicity conferring by typical HSP90 inhibitors^49^. **D6** might modulate HSP90-centered PPIs because **D6** is biased to constrain the interaction between HSP90 and T790M/L858R-EGFRs. Supporting this notion, **D6** was found to impair EGFR and HSP90 interaction in NCI-H1975 cells, noting that this interaction was unaffected in A549 and PC9 cells (Fig. 5f and Supplementary Fig. 23b, c). To exclude any cell-context-dependent effects, we recapitulated those experiments in the HEK293 cells. As shown, **D6** weakened the interaction between HSP90 and EGFR carrying T790M mutation; notably, this is the case with L858R/T790M and Del19/T790M-EGFR mutants, both of which mainly evolve to resist the first generation of EGFR-TKIs (Fig. 5g and Supplementary Fig. 23d). These results indicate that **D6** selectively promotes erlotinib-resistant EGFR degradation by jeopardizing HSP90 binding.

### D6 inhibits the growth of erlotinib/gefitinib-resistant NSCLC xenografts

**Fig. 6:**
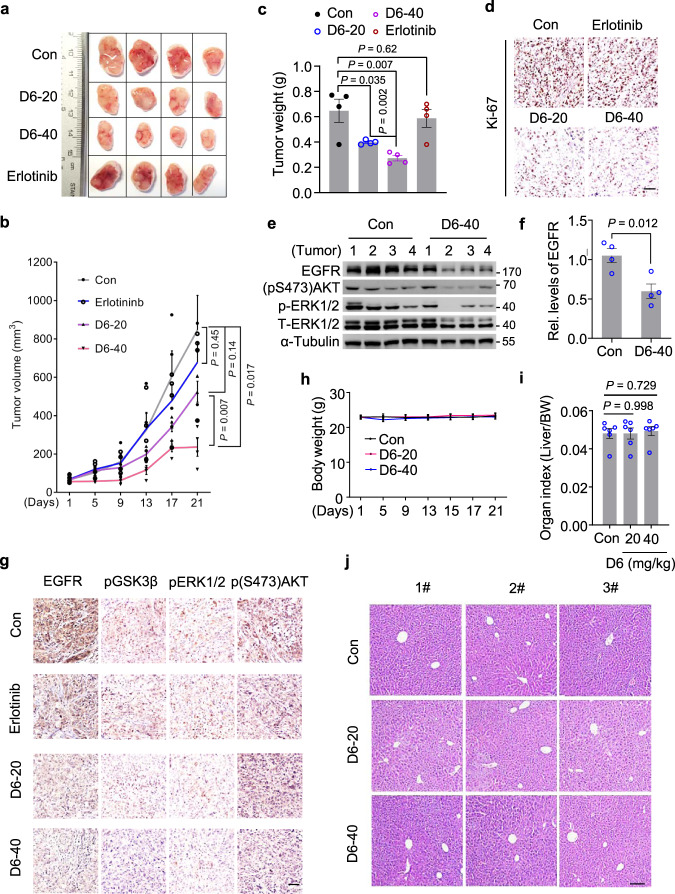
D6 inhibits the growth of xenografts developed from NCI-H1975 cells. **a** Nude mice inoculated with NCI-H1975 cells were randomly divided into four groups (*n* = 4 mice per group). When tumor volume reached ~100 mm^3^, mice were administered (i.p.) with **D6** (20 or 40 mg/kg) or erlotinib (100 mg/kg), respectively. The remaining mice injected with an equivalent volume of solvent were taken as control. After 21 days, mice were sacrificed and tumors were collected for analysis. **b**, **c** Tumor volume (**b**) and tumor weight (**c**) were analyzed in each group of mice. To calculate tumor volume, the tumor width and length were measured by a caliper. **d** Representative images showing the IHC staining of Ki-67 expression in tumors isolated from (**c**). Scale bar, 100 µm. **e**, **f** Immunoblotting analysis of protein lysates isolated from tumors in (**c**). EGFR expression levels were quantified (**f**), *n* = 4 mice for indicated groups. **g** Representative images showing the IHC staining of indicated proteins in tumors isolated from (**c**). Scale bar, 100 µm. **h−j** Mouse body weight (**h**), mouse liver organ index (**i**), and mouse liver histological morphology (**j**) were analyzed post-application with indicated dosages of **D6**. Con: solvent solution; D6-20 or D6-40: mice treated with D6 at the dose of 20 or 40 mg/kg. Data are presented as mean ± SEM, *n* = 4 mice (**b**, **c**, and **f**) or *n* = 6 mice (**i**). *P* values were calculated by two-way ANOVA analysis (**b**) or two-tailed Student’s *t*-test (**c**, **f**, and **i**).

We next asked whether **D6** could overcome T790M-EGFR-mediated TKI resistance. To investigate this, we generated isogenic erlotinib-resistant cells (Er-R) by stably expressing ectopic Del19/T790M-EGFR in the parental erlotinib-sensitive PC9 cells (Supplementary Fig. 24a). While Er-R cells showed a strong resistance to erlotinib (from 1000 to 5000 nM), **D6** at lower dose (2.5 µM) was sufficient to overcome such resistance (Supplementary Fig. 24b). We further evaluated the in vivo anti-tumor effects of **D6** on erlotinib-resistant NCI-H1975 cells expressing L858R/T790M EGFR. We generated xenografts by subcutaneous inoculation of NCI-H1975 cells in nude mice. After the tumors reached ∼100 mm^3^, mice were separated into four groups and treated (via i.p. injection) with **D6** (20 mg/kg and 40 mg/kg), erlotinib (100 mg/kg) and solvent once every 2 days, respectively. The volume of xenografts showed reductions of 44.4% or 77.8% in mice treated with **D6** at the dose of 20 or 40 mg/kg compared with solvent control; noting that erlotinib exerted no significant effect (Fig. 6a, b). **D6** reduced tumor burden and elicited a marked decrease of Ki-67 expression (Fig. 6c, d). In accordance with the effect of D6 on tumor regression, D6 therapy suppressed the EGFR signaling activity: determined by immunoblotting analysis of isolated xenografts, D6 suppressed EGFR expression and relevant downstream effectors, e.g., (pS473)AKT and pERK1/2 (Fig. 6e, f). This effect was further confirmed by immunohistochemical (IHC) staining analysis, showing that tumors in mice exposed to **D6** had a progressive decrease of EGFR protein, (pS473)AKT, phospho-ERK1/2 (pERK1/2), and (pS9)GSK3β (pGSK3β) (Fig. 6g). Notably, negligible toxicity of D6 was observed under such experimental conditions, as evidenced by little changes of the mouse body weight (Fig. 6h), the liver organ index (Fig. 6i), and the liver histological morphology (Fig. 6j). These results suggest the potential clinical significance of **D6** by targeting T790M-EGFRs in erlotinib/gefitinib-resistant NSCLCs.

### D6 has the potential to overcome the osimertinib resistance in NSCLC

**Fig. 7:**
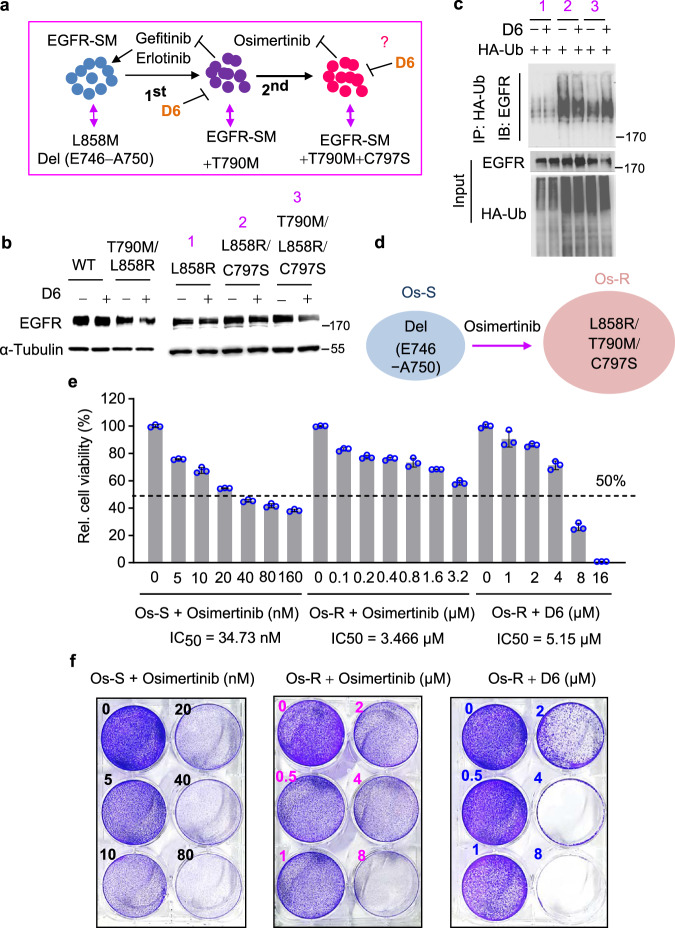
D6 overcomes osimertinib resistance in NSCLCs. **a** Schematic diagram illustrating the development of EGFR-TKI resistance. **b** Immunoblotting analysis showing the expression of indicated EGFR mutants transfected into HEK293 cells and treated with or without **D6** (2.5 µM). The numbers 1, 2, and 3 represented indicated EGFR mutants. **c** Cell lysates derived from HEK293 cells transfected with HA-Ub and indicated EGFR mutants with **D6** (2.5 μM) treatment or not were incubated with anti-HA beads. The elution of anti-HA beads was then subjected to immunoblotting analysis. **d** Schematic diagram showing the generation of osimertinib-resistant PC9 cells (Os-R) from the osimertinib-sensitive parental cells (Os-S). **e** Os-S or Os-R cells were treated with the indicated concentrations of osimertinib or **D6**. Cell viability was detected by CCK8 and displayed after normalized to the control. **f** The representative images showing the cell proliferation of Os-S or Os-R cells exposed to the indicated concentrations of osimertinib or **D6** for long-term culture in 6-well plate. Data are presented as mean ± SEM (**e**), *n* = 3 biologically independent samples.

Osimertinib is a representative third-generation EGFR-TKI and has been approved to treat gefitinib or erlotinib-resistant T790M-positive NSCLCs^15^. However, osimertinib-resistance mutation, such as the frequently identified C797S, frequently emerge within the first year of treatment^18,50^. As shown in Fig. 7a, tumor cells in some patients might evolve with multiple mutations including T790M, C797S, plus the original EGFR-TKI-sensitive mutations (SM: sensitive mutation, e.g., L858R or Del19 E746_A750) (Fig. 7a). Under such circumstances, applying EGFR-TKIs including osimertinib is ineffective. As **D6** destabilized T790M-EGFR mutants, we assessed if **D6** would achieve similar efficacy in osimertinib-resistant NSCLCs. As shown, **D6** downregulated the protein level of both L858R/T790M and L858R/T790M/C797S-EGFR but had little effect on L858R alone or L858R/C797S mutated forms (Fig. 7b). Consistently, **D6** greatly facilitated the ubiqutination of L858R/T790M/C797S-EGFR (Fig. 7c). These results suggest that D6 has the potential to treat osimertinib-resistant NSCLCs. To support that notion, we generated an osimertinib-resistant PC-9 cell line (Os-R) which was transfected with L858R/T790M/C797S-EGFR and then selected by osimertinib (Fig. 7d). In comparison with the parental PC-9 cells (Os-S) which were sensitive to osimertinib (IC_50_ = 34.73 nM), Os-R cells displayed apparent resistance (IC_50_ = 3.466 µM, ~100 folds higher) (Fig. 7e, left and middle). Importantly, **D6** achieved the potent inhibitory effect on Os-R cells at a low dose (IC_50_ = 5.15 µM) (Fig. 7e, right). As determined by cell proliferation assay, **D6** was detrimental to Os-R cell growth at the dose of 4 µM (Fig. 7f, right). By contrast, Os-R cells showed resistance to osimertinib even at the dose of 8 µM (Fig. 7f, middle). Altogether, these results indicate that **D6** may have the potential to treat the osimertinib-resistant NSCLCs.

## Discussion

Most NSCLC patients harboring EGFR-activating mutations have a high response rate and achieve a survival benefit when treated with the first generation of EGFR-TKIs, gefitinib, or erlotinib^4^. However, acquired resistance elicited by the secondary EGFR T790M mutation typically emerges within 9–13 months^10,13^. Here, we demonstrate that **D6** treatment reduces the viability of NSCLC cells harboring T790M-EGFR mutations that mediated TKI resistance in both in vitro and in vivo models. Particularly, **D6** treatment does not widely affect noncancerous cells, suggesting its low toxicity. At the molecular level, **D6** induces a fast degradation of TKI-resistant T790M-EGFR by jeopardizing its binding to HSP90 but has minor effect on the chaperone activity. Therefore, theoretically, **D6** treatment may lead to less side-effects in comparison with canonical HSP90 inhibitors. Our results provide an alternative strategy to overcome the T790M-EGFR mutant in NSCLCs based on **D6** treatment. Considering the further application of **D6**, it is crucial to analyze the systemic bioavailability in future studies.

There is a clear clinical need to overcome T790M-mediated resistance to EGFR-TKIs, and avenues to overcome this resistance are important to pursue. One approach is the development of the second generation of EGFR-TKIs that covalently modify EGFR, such as afatinib^12,14^. However, due to irreversible covalent binding, they potently suppress the kinase activity of both WT and mutated EGFR and lead to dose-limiting toxicities, which limits the therapeutic window. Alternatively, osimertinib is an oral and irreversible third generation of EGFR-TKI with potent and selective inhibition of T790M EGFR mutant over WT, which has been widely used for EGFR-T790M-positive NSCLC patients^15^. Unfortunately, resistance mutations also emerge over the course of treatment, most frequently observed as C797S^15–17^. In this study, we unravel that **D6** treatment displays a promising efficacy to eliminate the L858R/T790M/C797S-EGFR-expressing NSCLC cells which are resistant to osimertinib. Additionally, a complicated issue not being deeply explored in current study is the efficacy of **D6** when applied to NSCLC patients harboring C797S mutation in *cis* or *trans* with T790M-EGFRs. Firstly, we have demonstrated that the modified PC9 cells expressing C797S-EGFR in *cis* with L858R/T790M were feasible to be targeted by **D6** (Fig. 7). Furthermore, **D6** had little effect on L858R/C797S-EGFR protein expression (Fig. 7b). These results suggest that **D6** most likely hits EGFR mutants harboring T790M in *cis* with C797S but less likely manipulates the form in *trans*. Critically, considering that C797S-EGFR is still sensitive to quinazoline-based EGFR-TKIs, patients harboring T790M mutation in *trans* with C797S are expected to respond well to the traditional first generation of EGFR-TKI in combination with osimertinib^16^. Here, we highlight the clinical significance of D6 targeting the EGFR mutants of C797S in *cis* with T790M, as which displays resistance to almost all EGFR-TKIs applied alone or in combination. In future study, it is worthwhile to explore the efficacy of **D6** combining with various EGFR-TKIs to prevent the acquisition of TKI resistance.

Cancer cells are dependent on chaperone proteins to survive in acute stress environments, e.g., hypoxic and nutrient-starved microenvironments, which makes most solid cancers sensitive to HSP90 inhibition^47^. Indeed, it is reported that mutant T790M-EGFRs in NSCLCs are more sensitive to HSP90 inhibitors than the WT cells and HSP90 inhibitors have the potential to overcome EGFR-TKI resistance^22–24^. Several HSP90 inhibitors have been evaluated in early clinical trial phase^26,29,49,51,52^. Unfortunately, most HSP90 inhibitors unselectively block chaperone activity and thus have severe side-effects, e.g., hepatotoxicity, and are abandoned for further applications^53^. In our study, we discover a small-molecule **D6** targets HSP90 and damages HSP90-mediated stabilizing T790M-EGFRs without affecting other canonical clients like CDK4 and c-Raf. Mechanically, **D6** preferably disrupts the interaction of HSP90 and T790M-EGFRs, whilst showing minimal effect on WT EGFR or first-generation TKI-sensitive EGFR mutants. Therefore, compared with the canonical HSP90 inhibitors, **D6** might have less toxicity. Future study to analyze these effects in vivo is necessary to understand how these findings can be translated into clinical therapy.

How **D6** exactly manipulates the interaction of HSP90 and T790M-EGFRs is unclear without detailed structural analysis. Indeed, HSP90-mediated chaperone system is complicated as different HSP90 clients are proposed to have co-chaperones or effectors to facilitate correct folding^47,54^. We assume that T790M-EGFR, comparing to WT or other EGFR mutants, might have evolved some distinctive mechanisms to well cooperate with HSP90 and paired cofactors. For instance, the association of T790M-EGFR with HSP90 is more evident in comparison with non-T790M mutations^23^ and T790M-EGFR-expressing NSCLC cells are much more sensitive to HSP90 inhibitors^22,24^. Thus, there may exist a HSP90-centred PPI network which accounts for T790M-EGFR stabilization. Another clue to support this notion is found in cases where erlotinib-resistant NSCLC cells have developed a mysterious HSP90-associated chaperone system to stabilize L858R/T790M-EGFR: it requires the help of cytosolic PKM2^55^. Like **D6** treatment, PKM2 has little effect on the HSP90 chaperone activity over other clients but a strong impact on T790M-EGFR. Recently, targeting HSP90-PPI is being proposed as an alternative strategy to precisely suppress HSP90’s partial function, and is expected to have lower toxicity than conventional HSP90 inhibitors^56^. Several teams have successfully developed molecules which are applied to disrupt the PPI of HSP90-CDC37, HSP90-P23, HSP90-AHA1, and HSP90-HOP^49^. Herein, this study highlights the **D6** targeting the PPIs of HSP90-T790M-EGFR, which may be beneficial to overcome the clinically vital issue of EGFR-TKI resistance.

In summary, we characterize **D6** as a novel small-molecule compound that shows potent and selective inhibition of NSCLC cells with T790M-mediated EGFR-TKI resistance. Particularly, we emphasize that **D6** differs from current typical HSP90 inhibitors as it specifically disrupts the interaction of HSP90 and T790M-EGFR without affecting the chaperone activity, which may lead to reduced toxicities. **D6** may impact the T790M-EGFR-positive NSCLCs by targeting the PPIs of HSP90/T790M-EGFRs, and these findings pave the way for further research into how **D6** treatment may treat EGFR-TKI resistance in clinical setting.

## Methods

### Cell culture

HCC827, PC9, A549, H1299, LO2, and HEK293 cells were obtained from ATCC (American Type Culture Collection). NCI-H1975 and MRC5 cells were purchase from the cell bank of the Chinese Academy of Sciences (Shanghai, China). NCI-H1975, HCC827, and PC9 cells were maintained in RPMI 1640 (Gibco, USA) and A549, MRC5, LO2, and HEK293 in DMEM (Corning, USA) supplemented with 10% FBS (PAN-Biotech, Germany) and antibiotics (100 U/ml Penicillin-Streptomycin, Gibco) at 37 °C in an atmosphere of 5% CO_2_ humidified environment. All cell lines are regularly authenticated by checking cell morphology and growth rates. If mycoplasma is contamination free is verified by PCR analysis (TaKaRa, Cat. #6601, Japan).

### Reagents and antibodies

Erlotinib (HY-50896), 17-AAG (HY-10211), MG132 (HY-13259), Osimertinib (HY-15772) and Recombinant Human Epidermal Growth Factor (rHuEGF, HY-P7109) were purchased from MedChemExpress (MCE), USA. Cycloheximide (No.S7418) was from Selleckchem, USA. Antibodies against cleaved PARP (#9548), ERK1/2 (#4695), phospho-ERK1/2 (Thr202/Tyr204) (#9101), AKT (#4691), and phospho-AKT (Ser473) (#4060) were purchased from Cell Signaling Technology, USA. Antibodies against β-actin, GAPDH, and α-Tubulin were from Beyotime Biotechnology, China. Secondary antibody coupled to Alexa Fluor 488 dye was purchased from Thermo Fisher, USA. HRP-conjugated secondary antibodies used for western blotting were from Jackson Immuno Research Lab., USA. Full antibody details are available in Supplementary Table 1.

### Cell viability

Cells were seeded onto 96-well plates at a density of 5000 cells per well and were allowed to adhere for 24 h. Cells with different treatments were subjected to viability detection by using the Cell Counting Kit-8 (CCK-8, MCE) according to the manufacturer’s notifications. In brief, 10 μl CCK-8 working solution was added to each well. After incubation at 37 °C for 1–2 h, the absorbance was measured at 450 nm using a Microplate Reader (PerkinElmer, USA).

### Transwell migration assay

In all, 5 × 10^4^ cells pretreated with **D6** for 1 h were suspended in 200 µl FBS-free medium and placed in the upper chamber (8 µm pore size, Corning). The insert was then incubated in a 24-well plate with 500 µl 10% FBS-supplemented medium as chemoattractant. Then, 4–6 h later, the top membrane was swiped with cotton swabs to remove non-migrated cells, and the cells on the other side membrane were stained by 0.1% crystal violet solution. Lastly, cell number was counted and normalized to evaluate the relative migration ability.

### Cell proliferation assay

In all, 2 × 10^3^ cells were seeded onto a 6-well plate and then treated by indicated concentrations of **D6** or equal volume of DMSO solvent. After 7 days, cells were fixed by 4% PFA and stained by 0.1% crystal violet solution. For quantification, the dye was eluted by 33% acetic acid and measured at 570 nm by using a microplate reader^57^. Relevant cell proliferation rate is presented as relative cell density by normalizing to control.

### Cell wound-healing assay

Cells were seeded onto a 12-well plate and allowed to grow to full confluency in complete medium. After **D6** treatment for 4 h, cell monolayer was scratched with a 10 µl-pipette tip to generate the wound. Cell migratory ability was measured and analyzed by recording the cell closure at an indicated time point.

### RNA isolation and qRT-PCR

Cells were lysed in Trizol reagent RNAiso Plus (Takara, Japan) and the total RNA was isolated by standard protocol and then transcribed into cDNA using 5× Primescript^®^ RT Master Mix (Takara, Japan) according to the manufacturer’s instructions. Gene expression was determined by qRT-PCR analysis by using 2× SYBR^®^ Green Mix (Takara, Japan) on a Bio-Rad detection system (Bio-Rad). The primers used in this study are listed below. h*EGFR*-F: 5′- GGCACTTTTGAAGATCATTTTCTC-3′, h*EGFR*-R: 5′-CTGTGTTGAGGGCAATGAG-3′; h*XIAP*-F: 5′-AGTGGTAGTCCTGTTTCAGCATCA-3′, hXIAP-R: 5′-CCGCACGGTATCTCCTTCA-3′; h*BCL-2*-F: 5′-CATGCTGGGGCCGTACAG-3′, h*BCL-2*-R: 5′-GAACCGGCACCTGCACAC-3′; h*BCL-XL*-F:5′-TGCATTGTTCCCATAGAGTTCCA-3′, h*BCL-XL*-R: 5′-CCTGAATGACCACCTAGAGCCTT-3′; h*Survivin*-F:5′-TGCCTGGCAGCCCTTTC-3′, h*Survivin*-R: 5′-CCTCCAAGAAGGGCCAGTTC-3′.

### Protein extraction and immunoblotting

Cell lysate was prepared following RIPA buffer and then protein extracts resolved in 5× Laemmli sample buffer (100 mM Tris-HCl [pH6.8], 4% SDS, 20% glycerol and 1 mM DTT) were separated by SDS-PAGE and transferred to a PVDF membrane (Millipore, USA). After being blocked with 5% non-fat milk and incubated with the respective primary antibodies and HRP-conjugated secondary antibodies, immunoblotting images were collected on a Bio-Rad system (Bio-Rad, USA). The detailed information about antibodies is shown in Supplementary Table 1.

### Immunoprecipitation

Cells with indicated treatments were lysed in IP lysis buffer (200 mM NaCl, 20 mM Tris-HCl (pH 7.9), 5 mM MgCl_2_, 10% glycerol, 0.2 mM EDTA and 0.1% NP-40) supplemented with protease inhibitors (Roche cOmplete Protease Inhibitor, Swiss). After sonication and centrifugation, the clear supernatant was collected and incubated with respective antibodies at 4 °C for 4 h or overnight. Immunoprecipitates were washed with IP lysis buffer and further resolved in 1.5× Laemmli sample buffer for further western blotting analysis.

### Immunofluorescence staining

Cells seeded onto coverslips were firstly treated with D6 for 12 h and then fixed with 4% paraformaldehyde, permeabilized with 0.1% Triton X-100 and blocked with 1% BSA. The anti-EGFR antibody was incubated overnight and recognized by the secondary antibody conjugated with Alex-488 dyes. After nuclei stained with DAPI, cells were observed and photographed under a fluorescent microscope (Zeiss, Germany).

### Animals

Nude mice (BALB/c nu/nu) (5–6 weeks old) were purchased from the Shanghai Laboratory Animal Center (SLAC) and maintained under regular conditions (22 °C, 40–60% humidity, a 12 h light/12 h dark light cycle). For xenograft implantation, NCI-H1975 cells (1 × 10^6^ cells/0.1 ml per mouse) were injected into the right front axilla of mice (s.c.) and then were randomly separated into four groups. When tumor volume reached ~100 mm^3^, mice of each group were applied to D6 (20 or 40 mg/kg), erlotinib (100 mg/kg) or solvent by i.p. injection. Tumor volume was measured with clipper ruler every other day. At the end of the experiment, tumor-bearing mice were anaesthetized, and tumors were removed and weighed. Experimental animals were housed and handled in accordance with protocols approved by the Committee on the Use of Live Animals in Teaching and Research of Shenzhen University.

### Immunohistochemical staining

Tumors were fixed in 4% paraformaldehyde for 48 h. Following embedding in paraffin, 5 μm sections were coated on slides, deparaffinized, and rehydrated. After quenching endogenous peroxidase activity and blocking non-specific binding sites, slides were incubated at 4 °C overnight with indicated primary antibodies and then followed with a proper secondary antibody incubating for 1 h. Sections were observed and imaged under optical microscope (ZEISS, Germany). Detailed information about used antibodies is shown in Supplementary Table 1.

### Statistics and reproducibility

The results are based on at least three independent experiments or biologically independent samples. Data are presented as means ± SEM or SD as indicated in the figure legend. The statistical *P* values were determined by Student’s *t*-test or two-way ANOVA analysis as indicated in this article.

### Reporting summary

Further information on research design is available in the Nature Research Reporting Summary linked to this article.

## Supplementary information

Supplementary information.

Description of Additional Supplementary Files.

Supplementary Data 1.

Reporting summary.

## Data Availability

All the data that support this study are included in this published article, Supplementary information file, and Source Data file. All source data are included in Supplementary Data 1 and Supplementary Fig. 25.
