# Supplementary material for: A small-molecule compound D6 overcomes EGFR-T790M-mediated resistance in non-small cell lung cancer
Source: Commun Biol. 2021 Dec 13;4:1391. doi: 10.1038/s42003-021-02906-4 (PMC8668973; doi:10.1038/s42003-021-02906-4)
Supplement: Supplementary file 1 — Supplementary information. [file 42003_2021_2906_MOESM1_ESM.pdf]

Supplementary information

A small-molecule compound D6 overcomes EGFR-T790M-mediated resistance in non-small cell lung cancer

Xiaolong Tang[1,2,3*#], Lizhi Cheng[1,4#], Guo Li[5#], Yong-Ming Yan[4], Fengting Su[2], Dan-Ling Huang[4], Shuping Zhang[5], Zuojun Liu[1,2,3], Minxian Qian[1,2], Ji Li[5], Yong-Xian Cheng[1,4*], Baohua Liu[1,2,3,6*]

[1]Guangdong Key Laboratory of Genome Stability and Human Disease Prevention, Shenzhen University, Shenzhen, China.

[2]Shenzhen Key Laboratory for Systemic Aging and Intervention (SAI), School of Basic Medical Sciences, Shenzhen University, Shenzhen, China.

[3]Shenzhen Bay Laboratory, Shenzhen, China.

[4]Institute for Inheritance-Based Innovation of Chinese Medicine, School of Pharmaceutical Sciences, Shenzhen University, Shenzhen, China.

[5]Department of Dermatology, Xiangya Hospital, Central South University, Changsha, China.

[6]Marshall Laboratory of Biomedical Engineering; International Cancer Center; Shenzhen University, Shenzhen, China.

[#]These authors contributed equally.

*Correspondence should be addressed to Dr Baohua Liu (ppliew@szu.edu.cn), Dr Yong-Xian Cheng (yxcheng@szu.edu.cn), or Dr Xiaolong Tang (tangxiaolong05@126.com).

This file contains Supplementary Figures 1-25, Supplementary table 1 and Supplementary methods.

Supplementary Figures

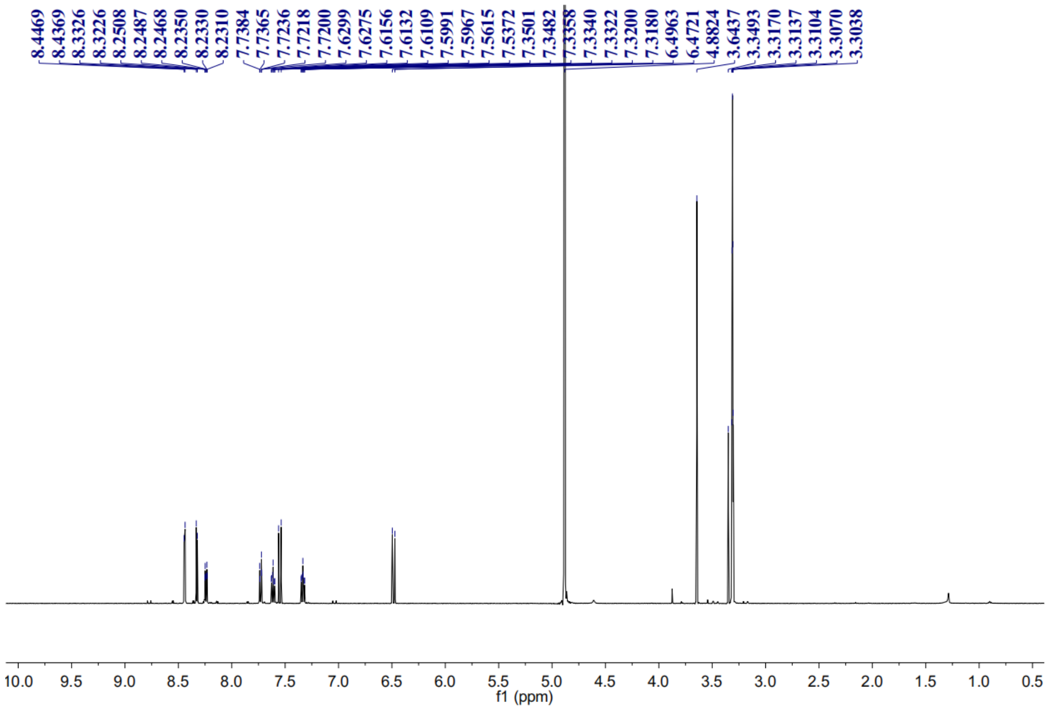

Supplementary Fig. 1 ^{1}H NMR (600 MHz) spectrum of D6-1.

The result showed the ^{1}H NMR (600 MHz) spectrum of D6-1 in MeOD.

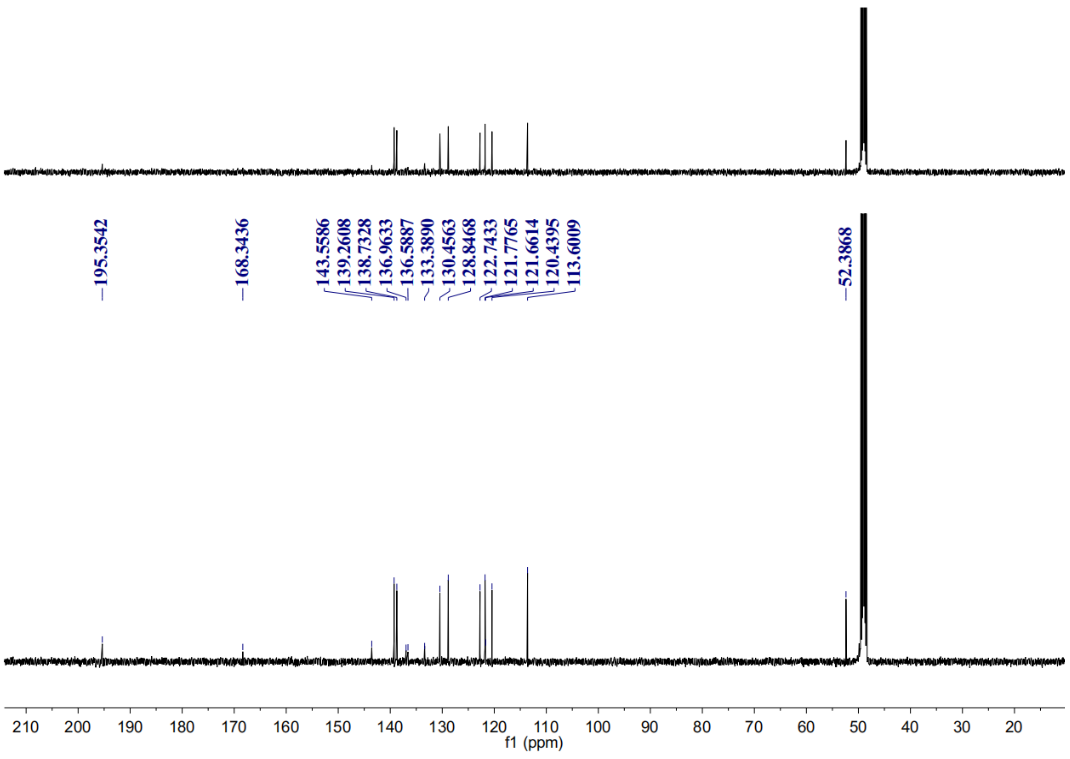

Supplementary Fig. 2 ^{13}C NMR (150 MHz) spectrum of D6-1.

The result showed the ^{13}C NMR (150 MHz) spectrum of D6-1 in MeOD.

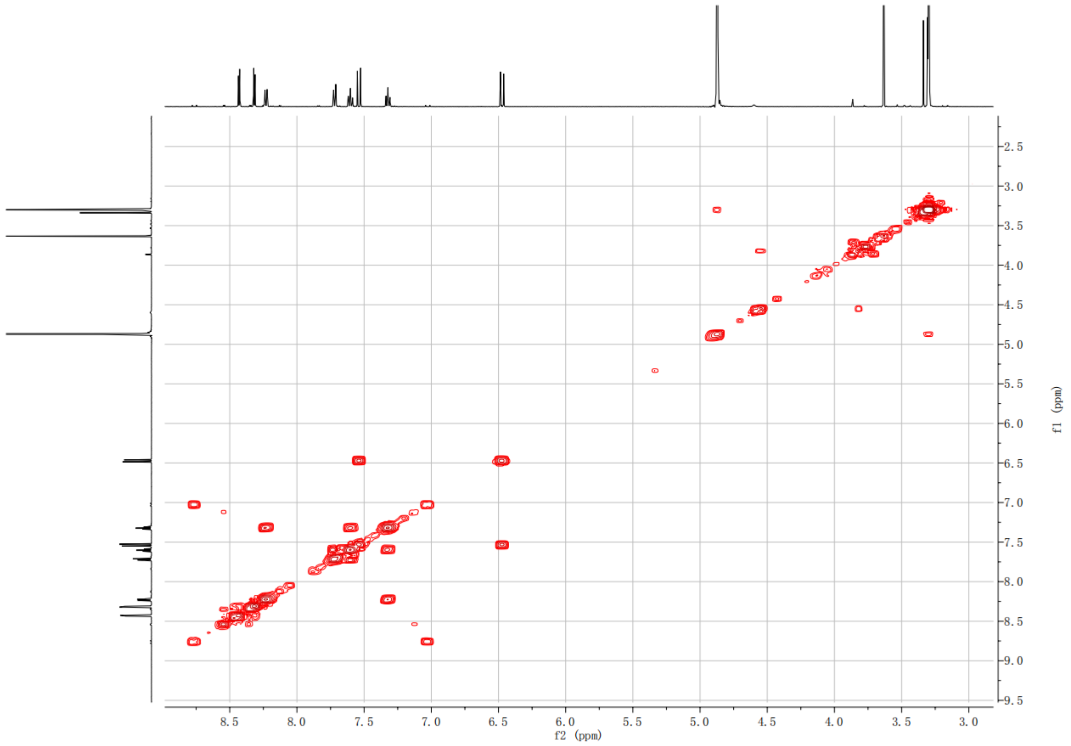

Supplementary Fig. 3 ^{1}H-^{1}H COSY (600 MHz) spectrum of D6-1.

The ^{1}H-^{1}H COSY (600 MHz) spectrum of D6-1 in MeOD was analyzed.

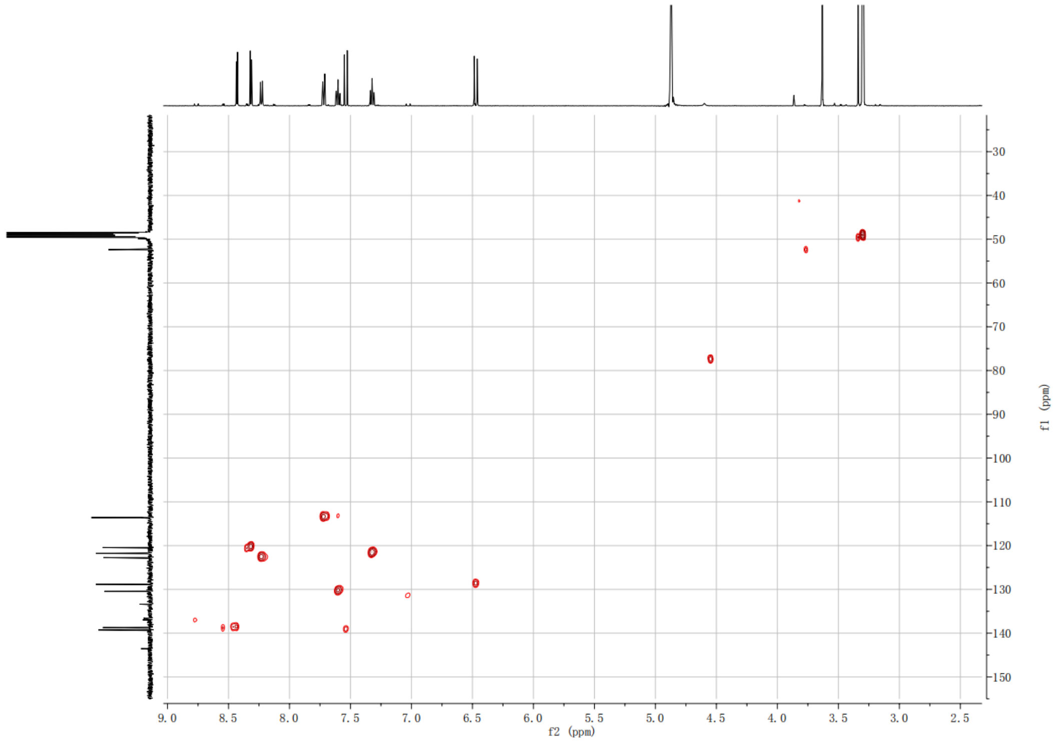

Supplementary Fig. 4 HSQC (600 MHz) spectrum of D6-1.

The HSQC (600 MHz) spectrum of D6-1 in MeOD was analyzed.

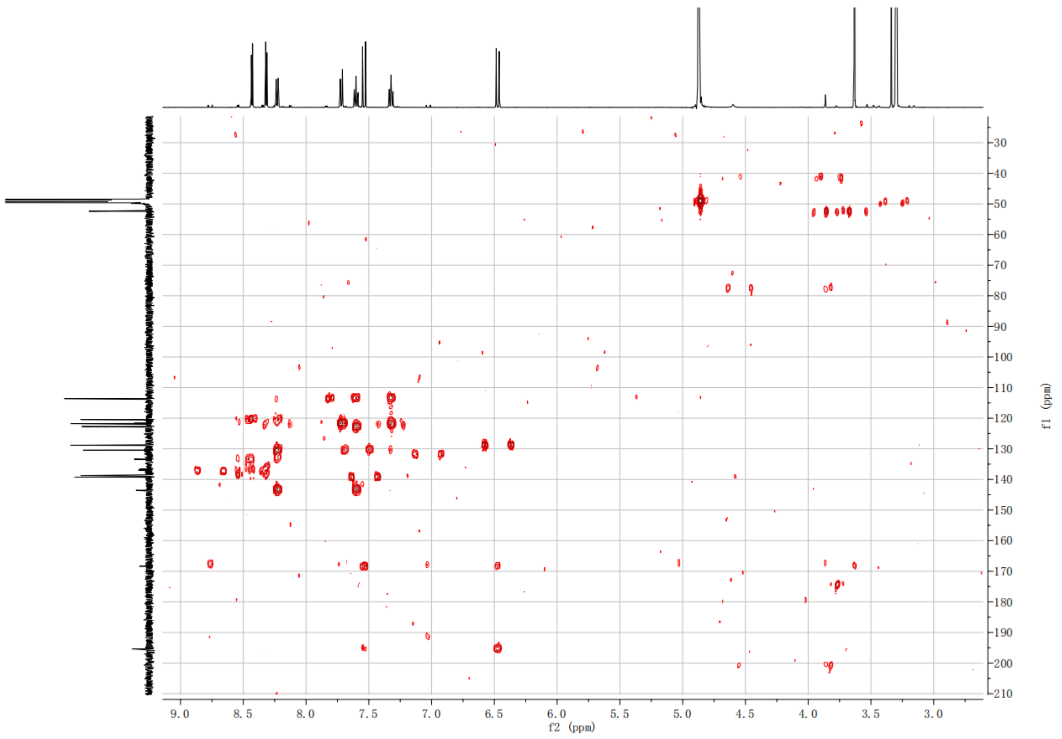

Supplementary Fig. 5 HMBC (600 MHz) spectrum of D6-1.

The data showed the HMBC (600 MHz) spectrum of D6-1 in MeOD.

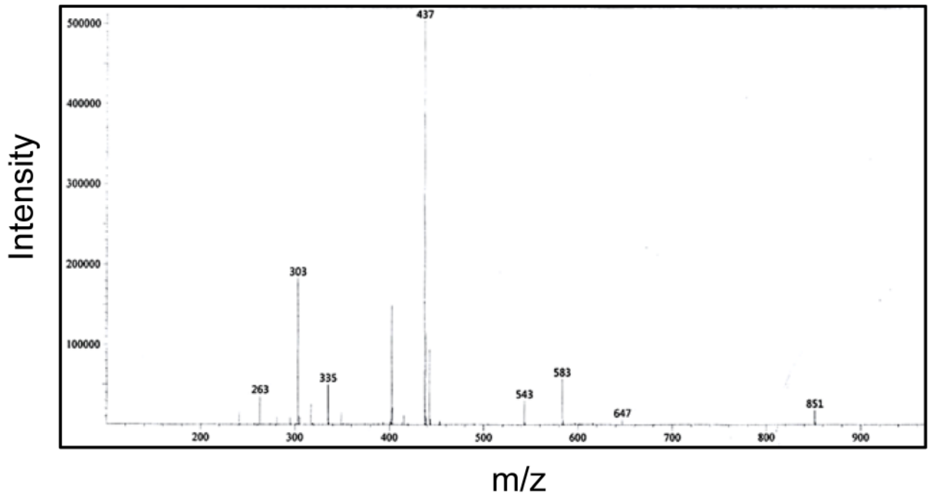

Supplementary Fig. 6 MS of D6-1.

The result showed the analysis of D6-1 by MS.

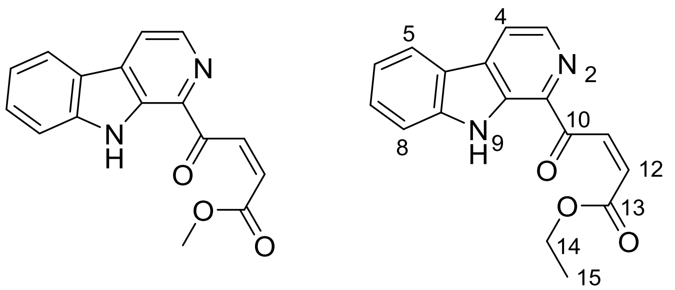

D6-1 **D6**

Supplementary Fig. 7 structures of D6-1 and D6.

Images showed structures of D6-1 and D6.

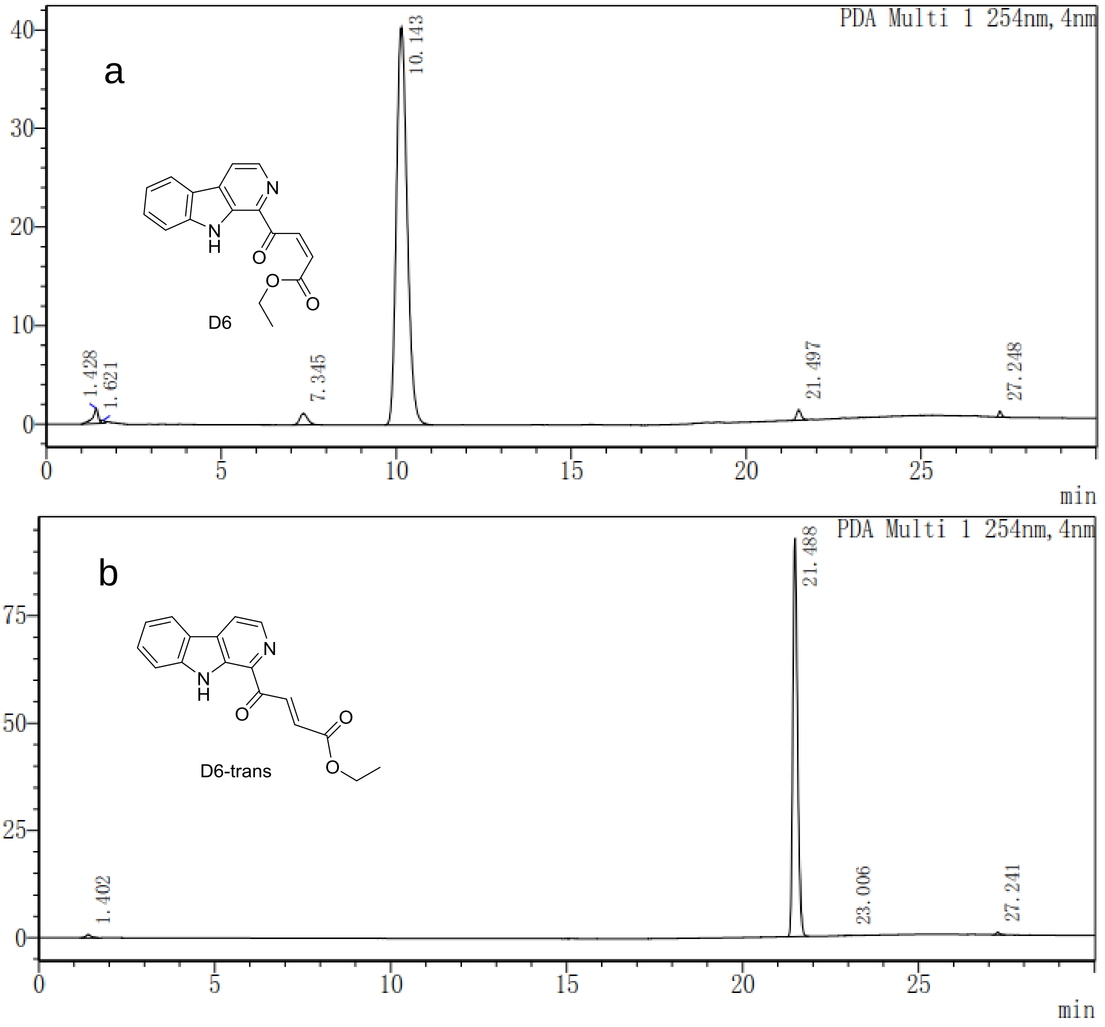

Supplementary Fig. 8 Purity check of D6 and D6-trans by HPLC.

(a) HPLC analysis of D6; (b) HPLC analysis of **D6-trans**. HPLC analysis method: Agilent E clipse XDB-C18, 5 μm, 4.6*150 mm, 0–15 min, MeOH/H_2O, 45%, 15–25 min, MeOH/H_2O, 45%–100%, and flow rate: 1 mL/min).

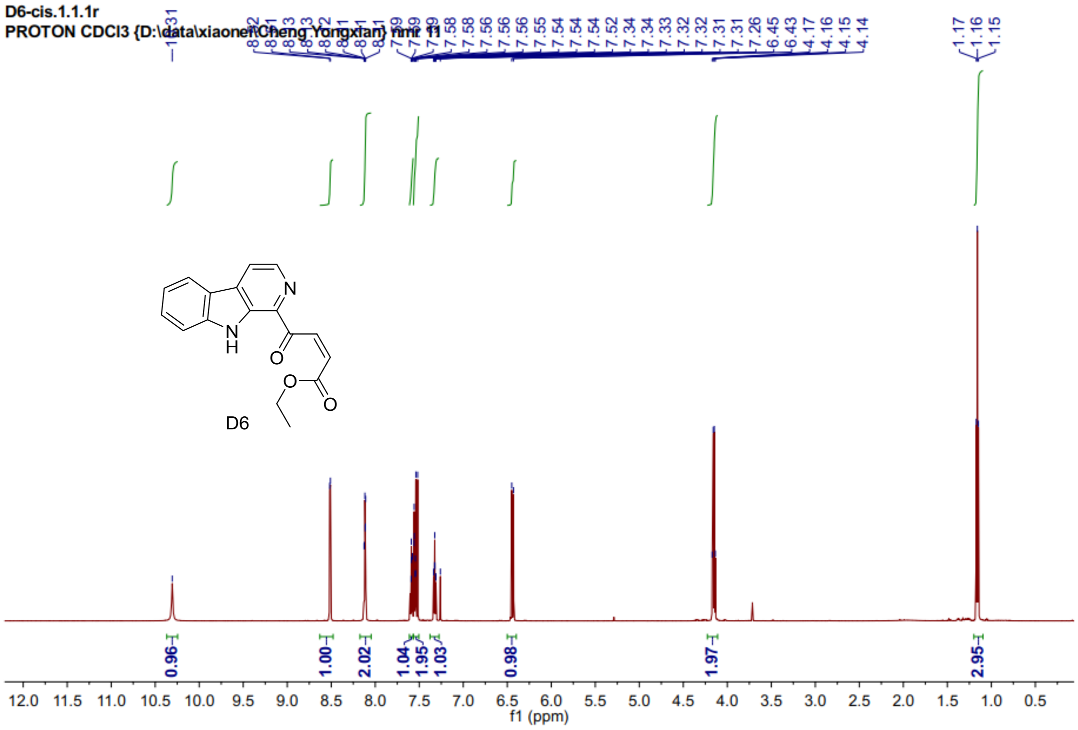

Supplementary Fig. 9 ^{1}H NMR (600 MHz) spectrum of D6.

^{1}H NMR (600 MHz) spectrum of **D6** in $CDCl_3$ was analyzed.

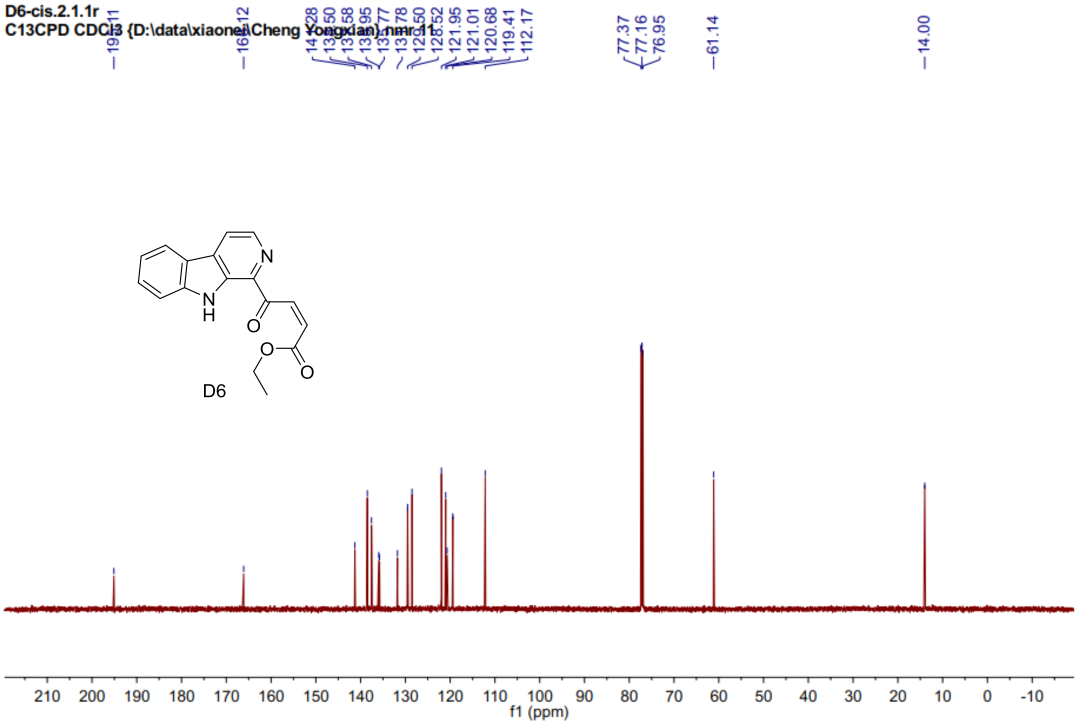

Supplementary Fig. 10 ^{13}C NMR (150 MHz) spectrum of D6.

^{13}C NMR (150 MHz) spectrum of **D6** in $CDCl_3$ was analyzed.

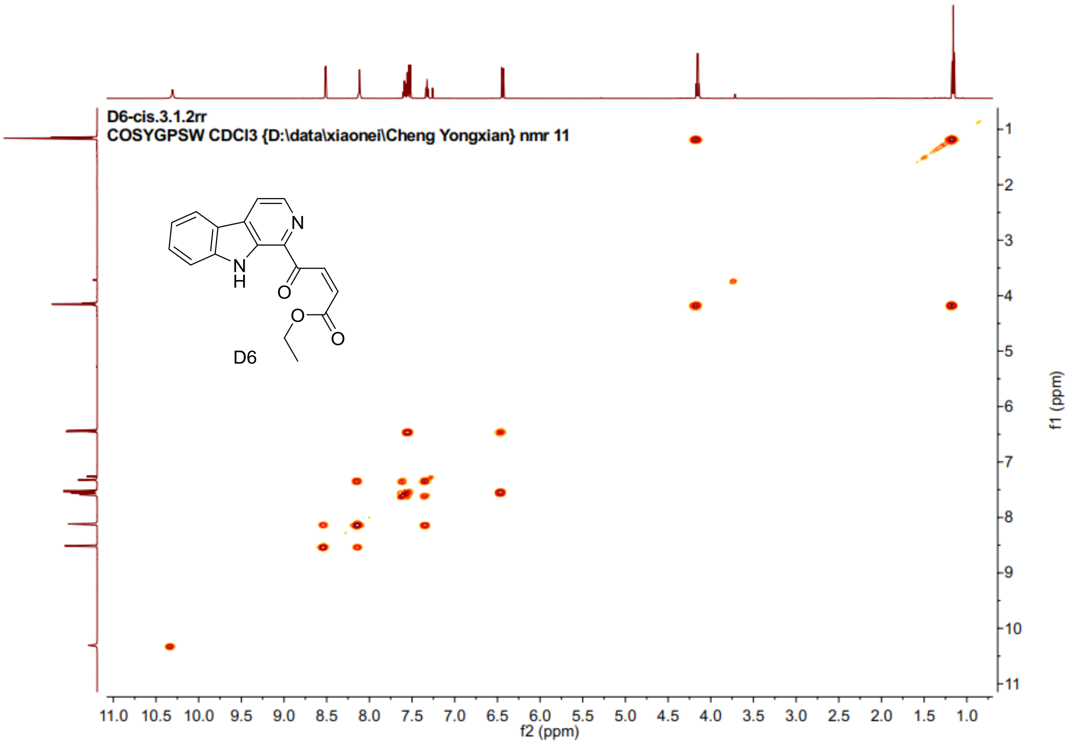

Supplementary Fig. 11 ^{1}H-^{1}H COSY (600 MHz) spectrum of D6.

The result showed the analysis of ^{1}H-^{1}H COSY (600 MHz) spectrum of **D6** in $CDCl_3$.

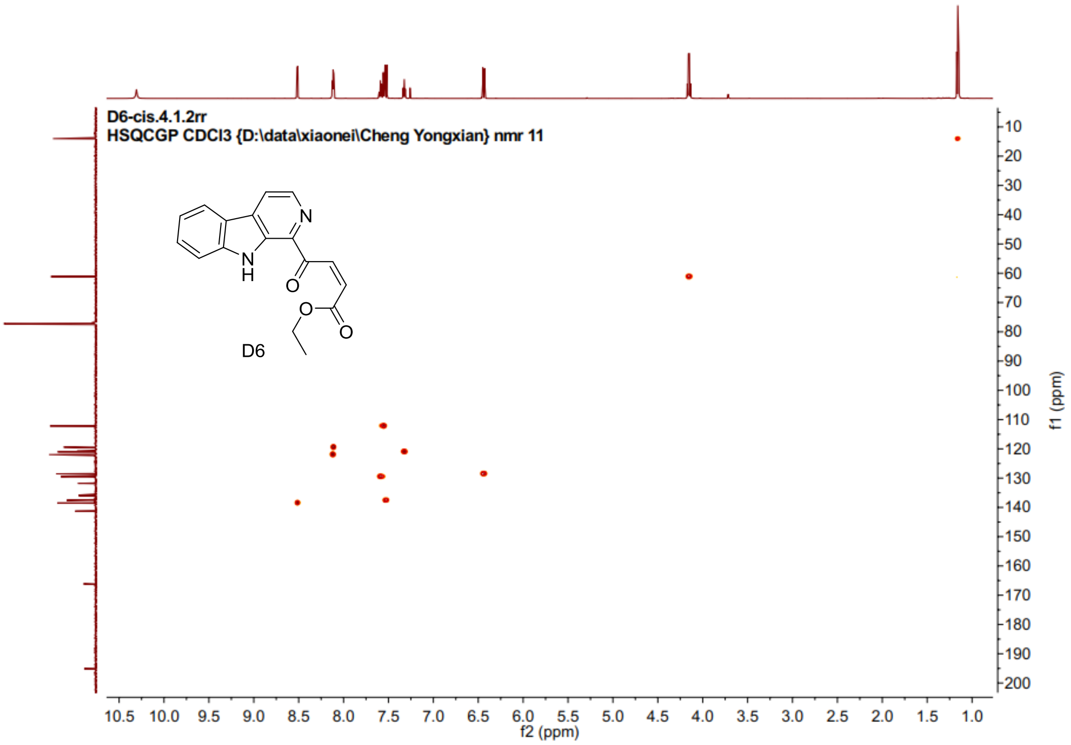

Supplementary Fig. 12 HSQC (600 MHz) spectrum of D6.

The HSQC (600 MHz) spectrum of **D6** in $CDCl_3$ was analyzed.

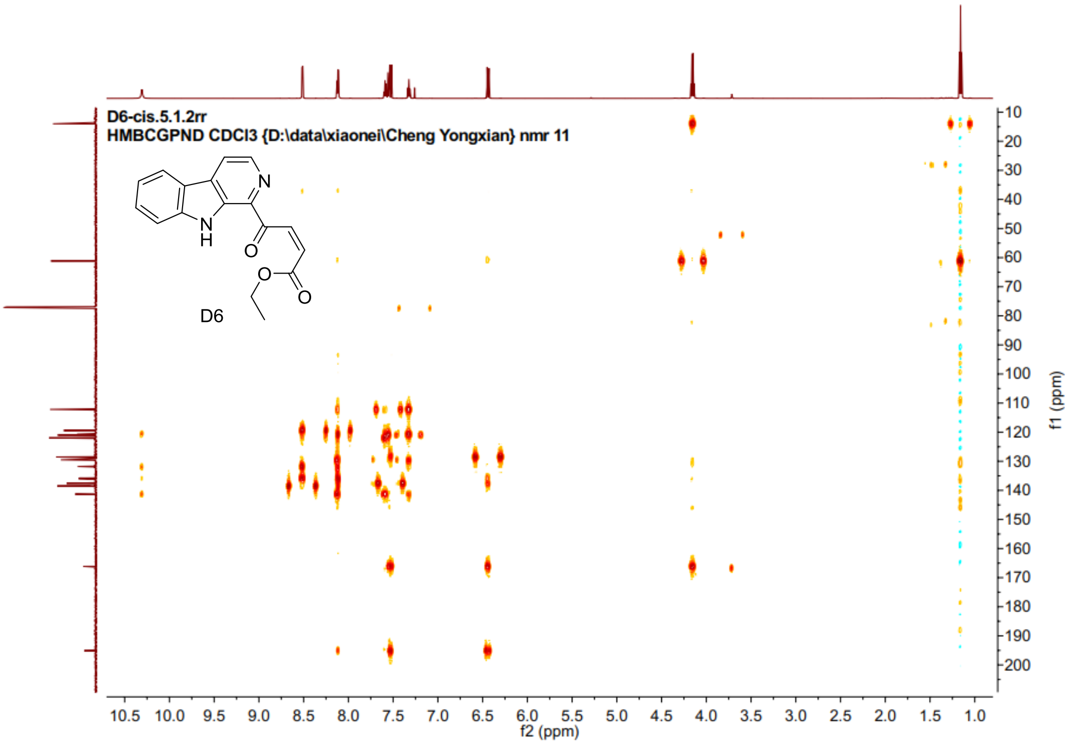

Supplementary Fig. 13 HMBC (600 MHz) spectrum of D6.

The HMBC (600 MHz) spectrum of **D6** in $CDCl_3$ was analyzed.

Created with SCIEX OS 1.4.1
Printed:4/12/2020 5:19:44 PM

Acquisition Date	4/12/2020 4:56:51 PM	Result Table	D6
Acquisition Method	N/A	Algorithm Used	AutoPeak
Project	N/A	Instrument Name	X500 QTOF

Mass Spretra

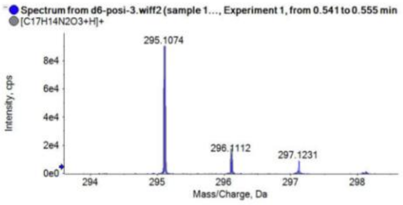

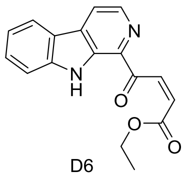

D6

#	Analyte Peak Name	Formula	Precursor Mass	Found At Mass	Mass Error (ppm)
1	D6 (0.600)	C17H14N2O3	295.1080	295.1074	-0.9

Supplementary Fig. 14 HRESIMS of D6.

The result showed the HRESIMS of **D6.**

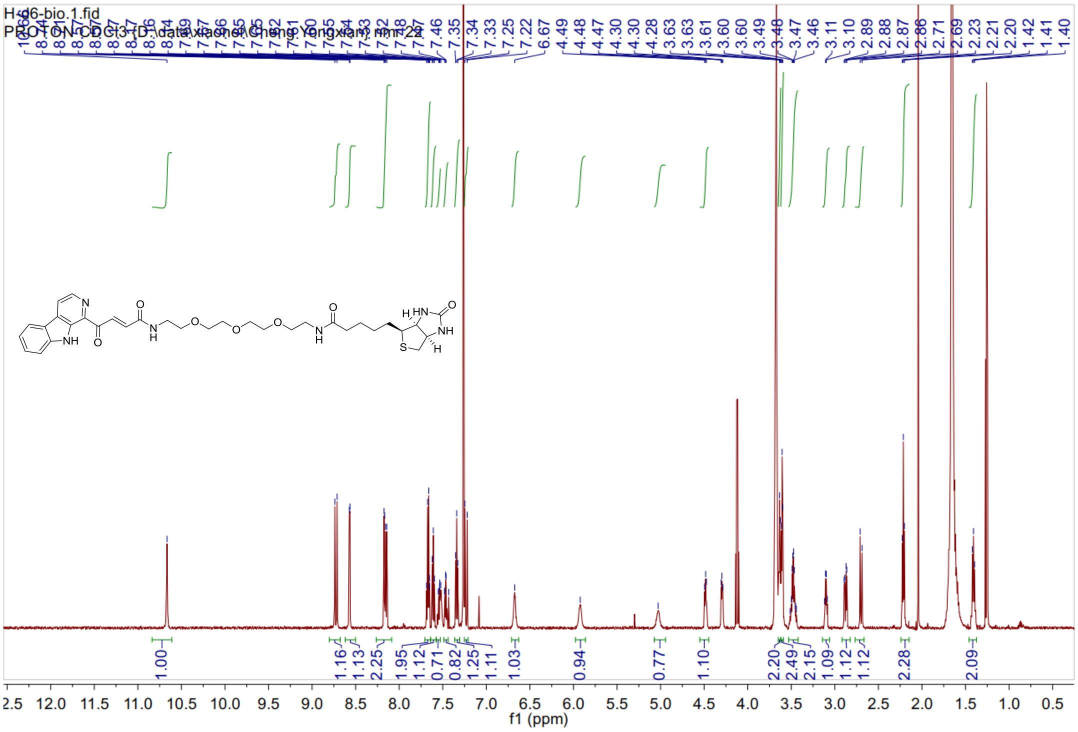

Supplementary Fig. 15 ^{1}H NMR (600 MHz) spectrum of D6-4.

The ^{1}H NMR (600 MHz) spectrum of **D6-4** in $CDCl_3$ was presented.

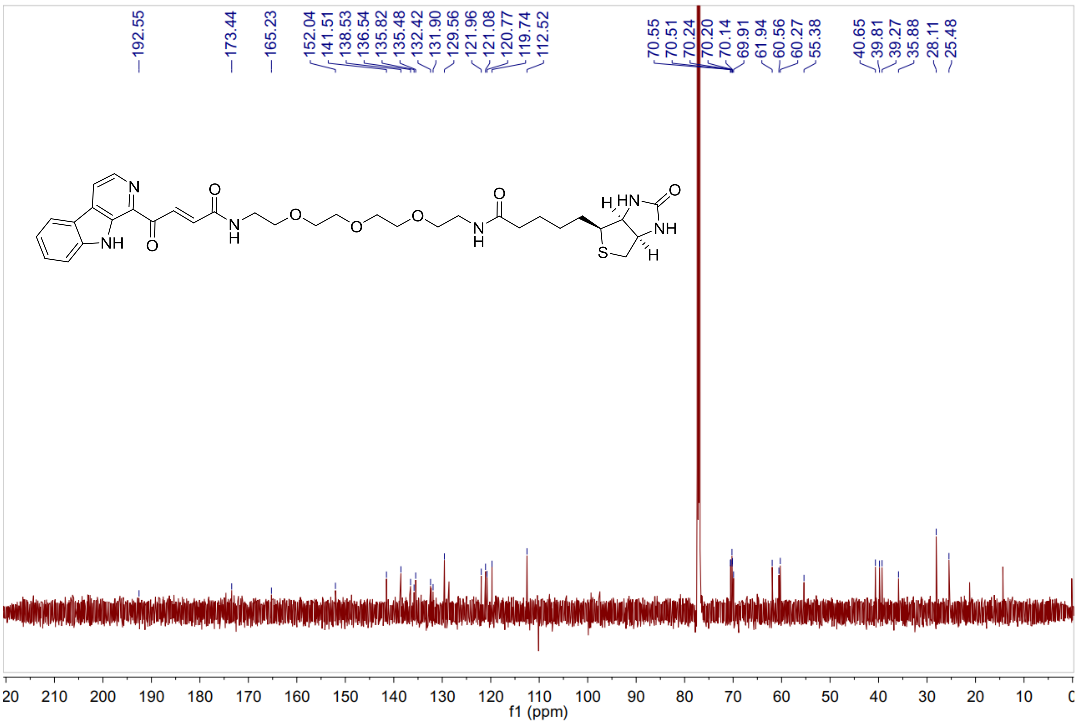

Supplementary Fig. 16 ^{13}C NMR (150 MHz) spectrum of D6-4.

The data represented the ^{13}C NMR (150 MHz) spectrum of **D6-4** in $CDCl_3$.

Created with SCIEX OS 1.4.1
Printed:5/12/2020 10:24:45 AM

Acquisition Date	5/12/2020 9:09:08 AM	Result Table	D6-BIO
Acquisition Method	N/A	Algorithm Used	AutoPeak
Project	HDL	Instrument Name	X500 QTOF

Mass Spretra

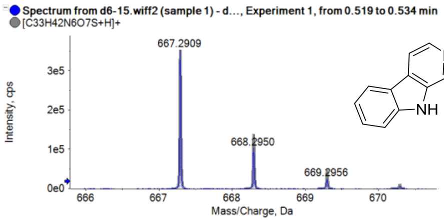

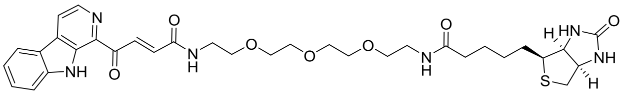

#	Analyte Peak Name	Formula	Precursor Mass	Found At Mass	Mass Error (ppm)
1	**D6-BIO**	C33H42N6O7S	667.2910	667.2909	0.1

Supplementary Fig. 17 HRESIMS of D6-4.

The presented data showed the HRESIMS of **D6-4.**

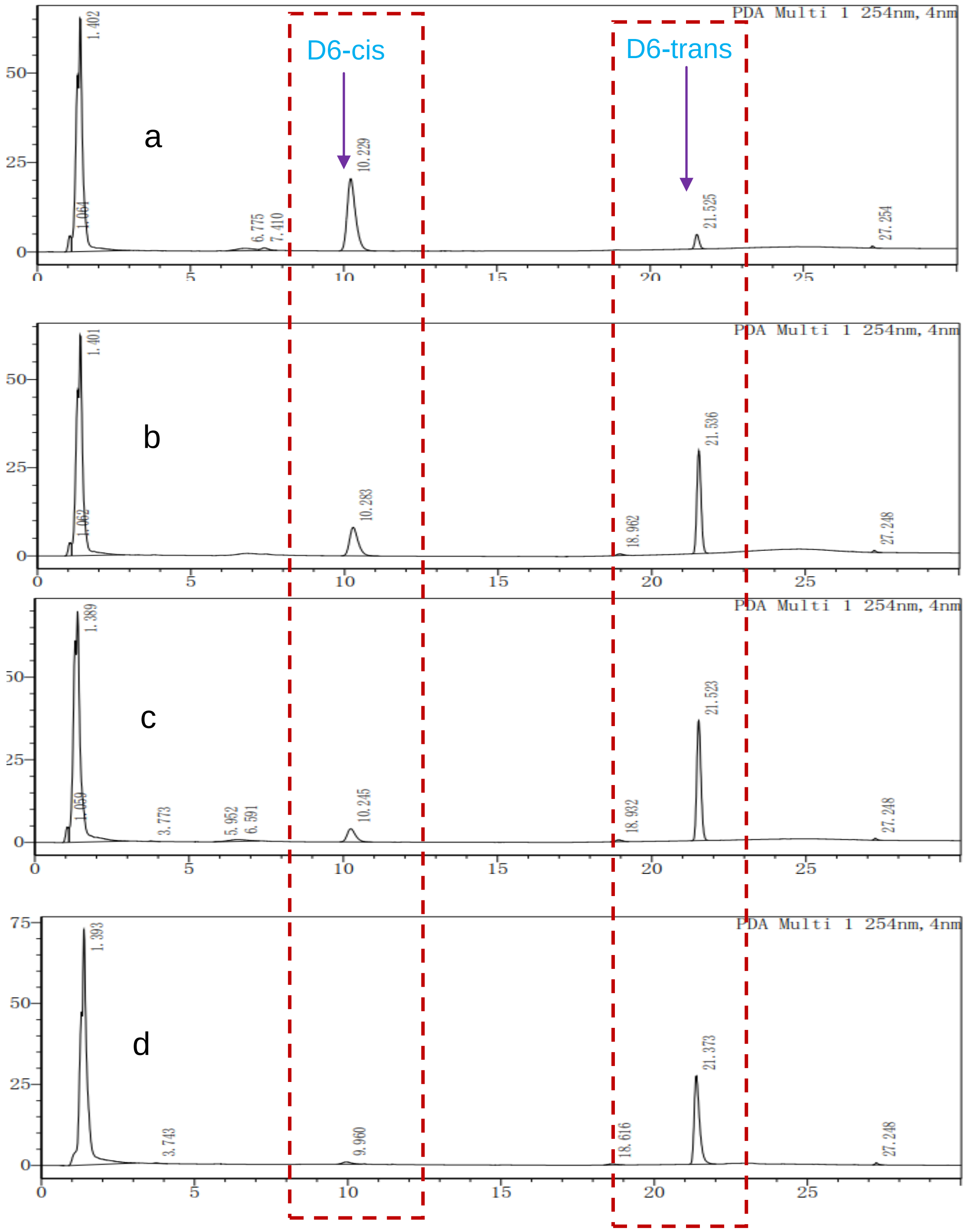

Supplementary Fig. 18 Analysis of D6 in cell culture medium by HPLC chromatogram.

(**a**) **D6** in cell culture medium at 0 h; (**b**) **D6** in cell culture medium at 6 h; (**c**) **D6** in cell culture medium at 12 h; (**d**) **D6** in cell culture medium at 24 h. HPLC analysis method: (Agilent E clipse XDB-C18, 5 μm, 4.6*150 mm, 0–15 min, MeOH/H_2O, 45%, 15–25 min, MeOH/H_2O, 45%–100%, flow rate: 1 mL/min).

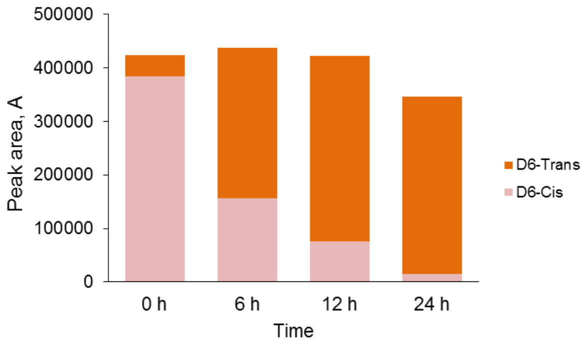

Supplementary Fig. 19 D6-Cis transits to D6-Trans.

HPLC chromatogram analysis of D6 diluted in cell culture medium at 0, 6, 12, and 24 h, respectively.

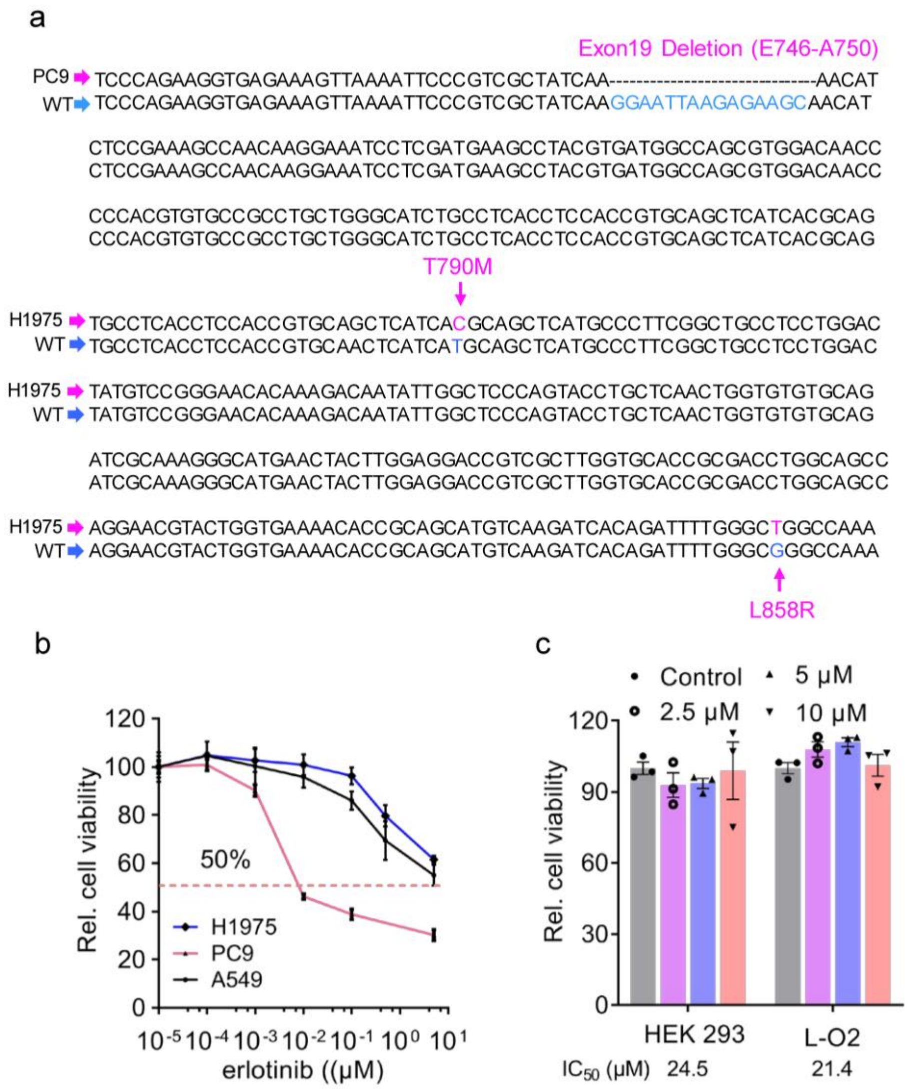

Supplementary Fig. 20 Analysis of EGFR status in 3 NSCLC cell lines.

(a) Genomic DNA sequencing to verify the EGFR status in A549, PC9 and NCI-H1975 cells. (b) PC9, A549 and NCI-H1975 cells were treated with a range of concentrations of erlotinib for 72 h. Cell viability is shown as % of relative viability by normalizing to the control. (c) Noncancerous HEK293 and L-O2 cells were treated with the indicated doses of **D6**. Cell viability was determined by CCK8 assay.

The bars and curves represent the means ± s.d. *P* values were calculated by student's *t*-test.

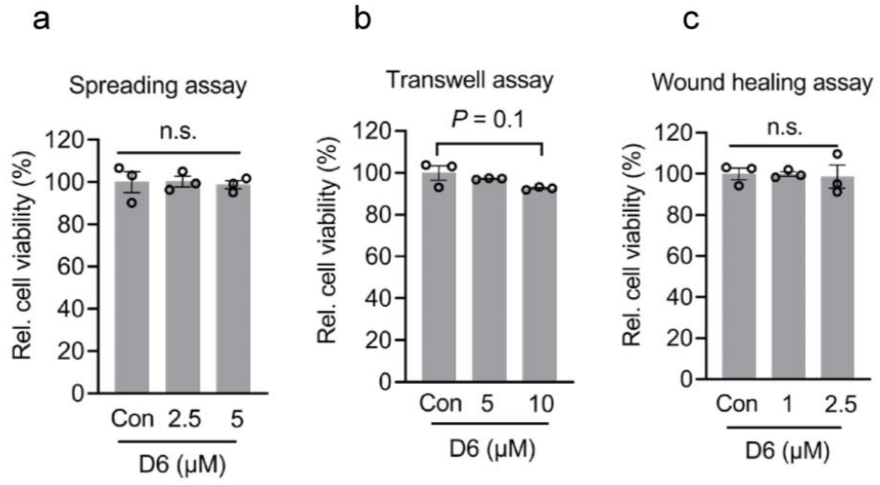

Supplementary Fig. 21 The effect of D6 on cell viability.

(a) H1975 cells pretreated by D6 for 4 hours and then cell viability was analyzed by CCK8 assay; n.s., no significance. (b) H1975 cells post-D6 application for 6 hours were subjected into viability analysis by CCK8 assay. (c) H1975 cells exposed to indicated dosages of D6 for 24 hours were employed for viability analysis by CCK8 assay; n.s., no significance.

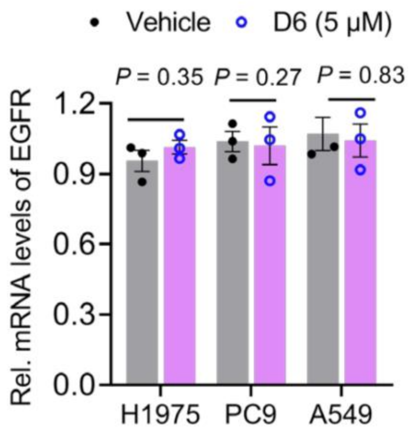

Supplementary Fig. 22 D6 affects little on *EGFR* mRNA expression.

Quantitative PCR analysis of *EGFR* mRNA levels in NCI-H1975, PC9 and A549 NSCLC cells treated with the indicated concentrations of **D6**.

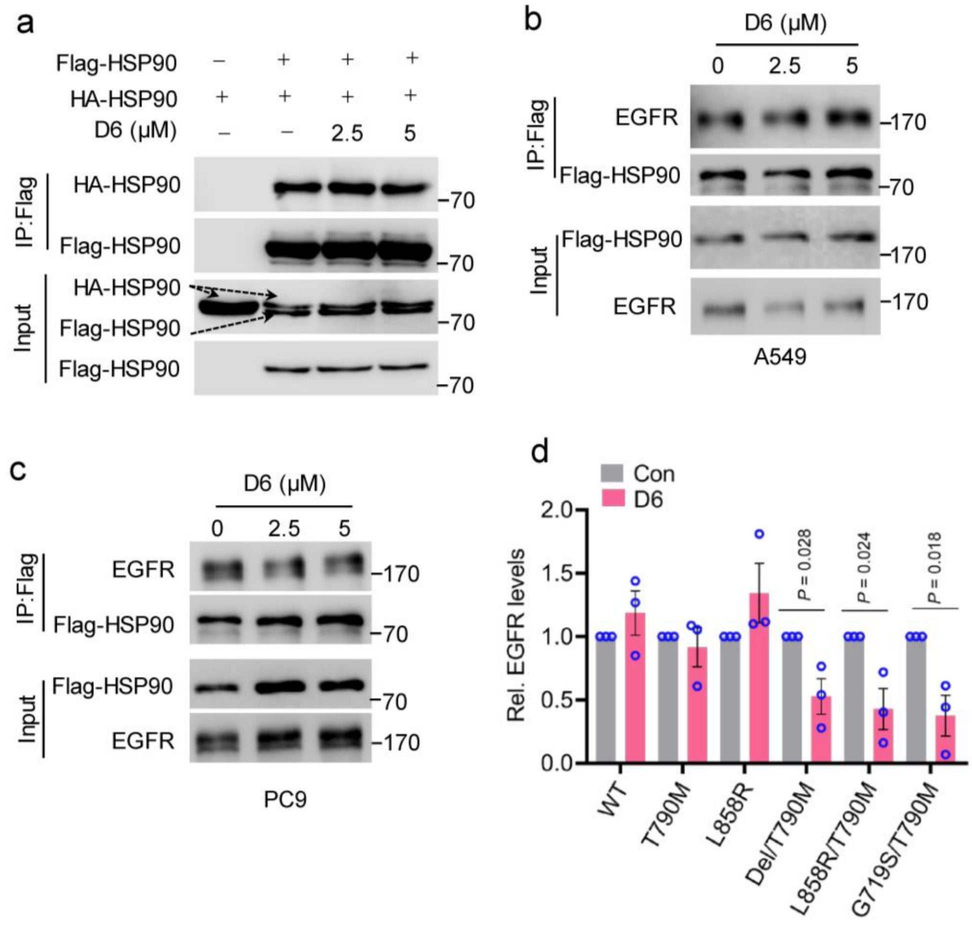

Supplementary Fig. 23 D6 uniquely disrupts the interaction of HSP90 and TKI-resistance EGFRs.

(a) Immunoblot analysis of Flag-HSP90 immunoprecipitates in HEK293 cells transfected with HA-HSP90 and Flag-HSP90 with or without the addition of **D6**. (b–c) Immunoblot analysis of anti-HSP90 immunoprecipitates derived from cellular lysates of A549 or PC9 cells treated with the indicated concentrations of **D6**. (d) Relative EGFR levels quantified from Figure 5g.

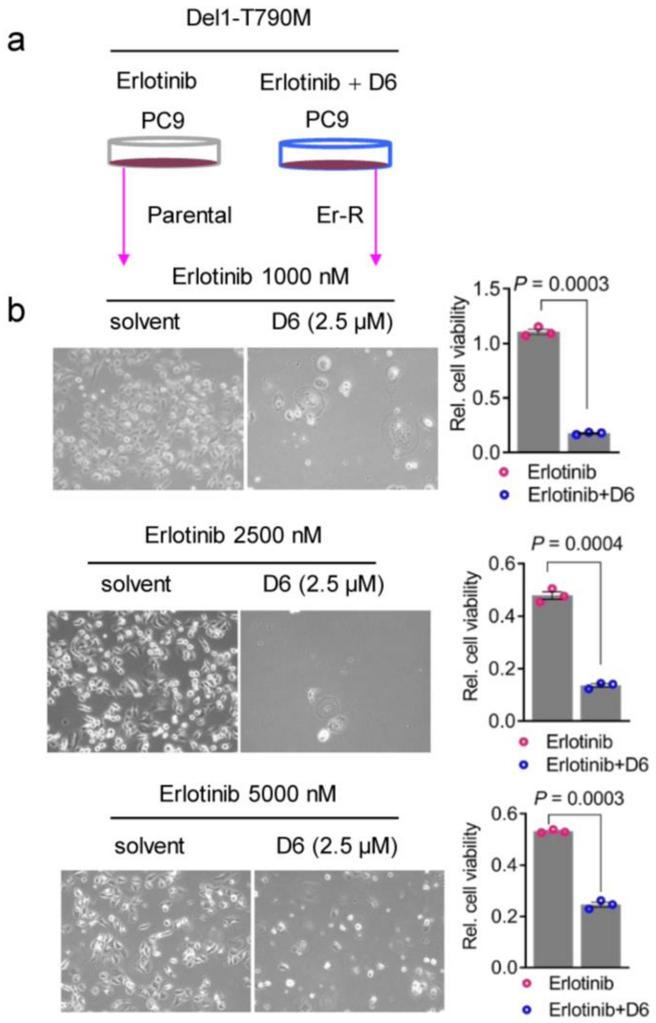

Supplementary Fig. 24 D6 has potentials to eliminate the erlotinib-resistance NSCLC.

(a) Schematic showing the generation of isogenic erlotinib-resistance PC9 cells (Er-R) by ectopically expressing Del1-T790M followed by erlotinib selection (see Methods for more details). (b) Representative images showing the growth of Er-R cells after treatment with the indicated concentrations of erlotinib or D6. Cell viability was measured by CCK8.

Supplementary Fig. 25 Uncropped blots

These uncropped blots are presented as the source data of Fig. 1f, Fig. 2d, Fig. 3a-c, Fig. 3e-f, Fig. 4a-b, Fig. 4d-e, Fig. 4g, Fig. 5b-g, Fig. 6e, Fig. 7b-c and supplementary Fig. 17b-c in the manuscript.

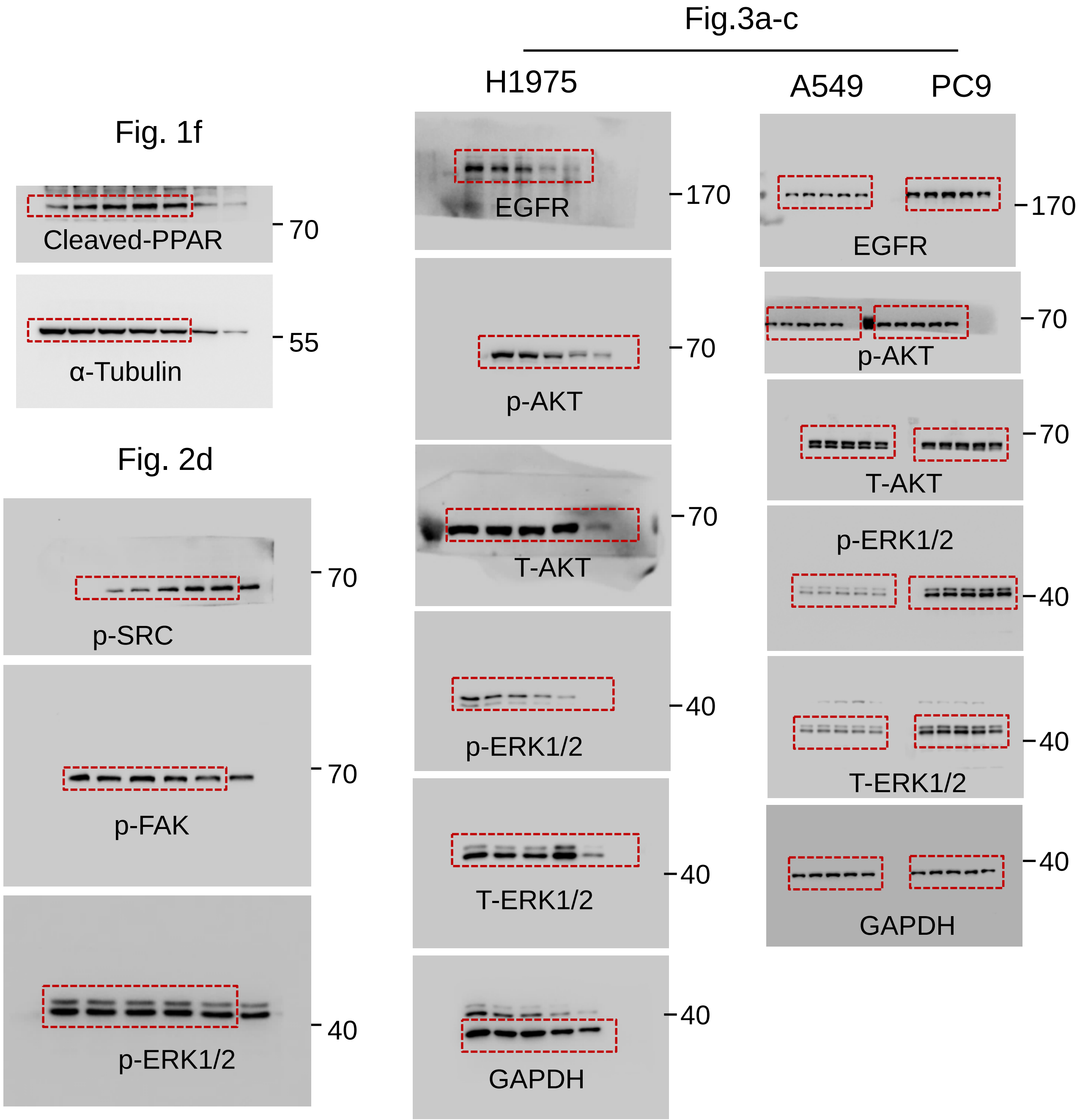

Fig.3a-c
H1975
A549
PC9
Fig. 1f
Cleaved-PPAR
70
α-Tubulin
55
Fig. 2d
p-SRC
70
p-FAK
70
p-ERK1/2
40
EGFR
170
p-AKT
70
T-AKT
70
p-ERK1/2
40
T-ERK1/2
40
GAPDH
40
EGFR
170
p-AKT
70
T-AKT
70
p-ERK1/2
40
T-ERK1/2
40
GAPDH
40

Fig. 3e

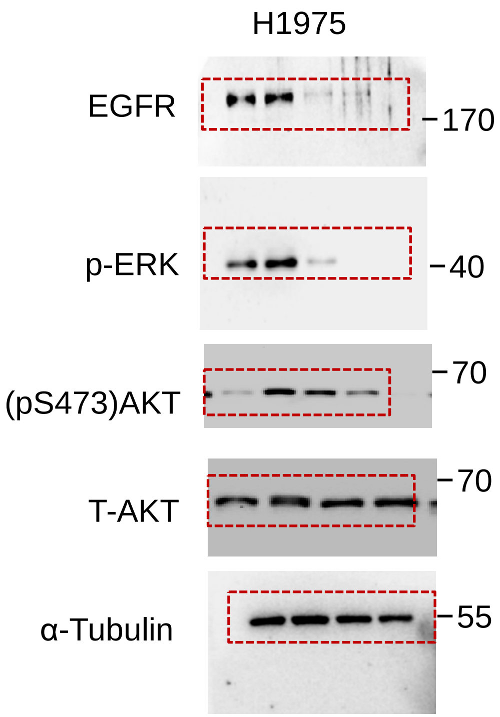
H1975
EGFR
170
p-ERK
40
(pS473)AKT
70
T-AKT
70
α-Tubulin
55

Fig. 3f

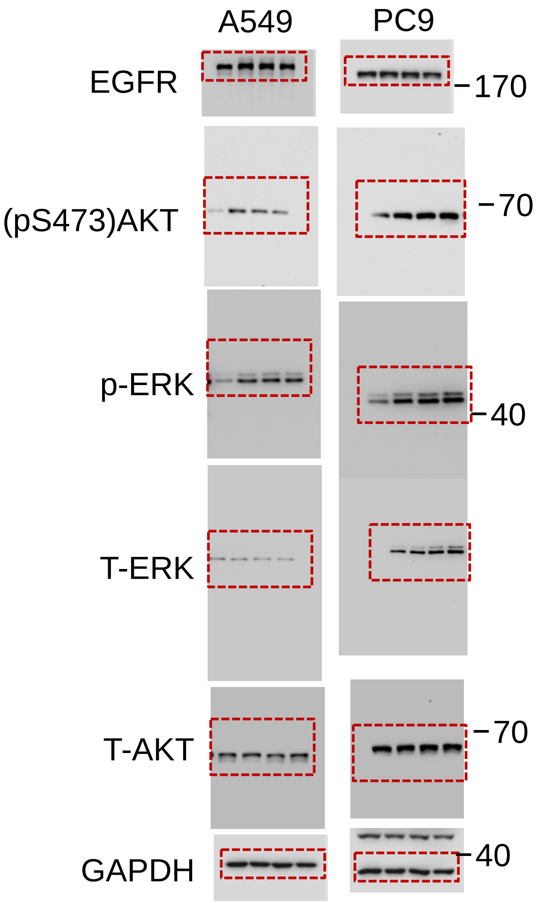
A549
PC9
EGFR
170
(pS473)AKT
70
p-ERK
40
T-ERK
T-AKT
70
GAPDH
40

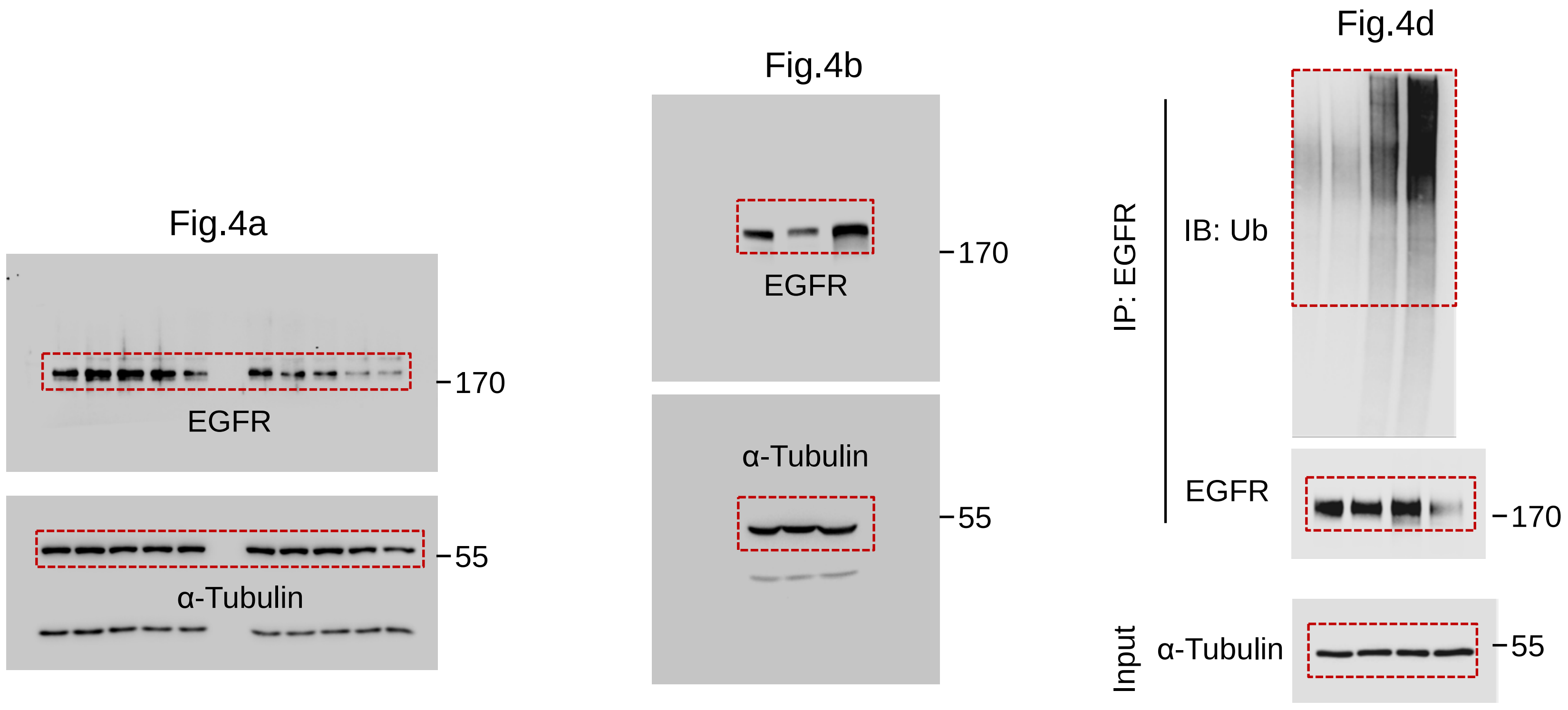
Fig.4a
EGFR
170
α-Tubulin
55
Fig.4b
EGFR
170
α-Tubulin
55
Fig.4d
IP: EGFR
IB: Ub
EGFR
170
Input
α-Tubulin
55

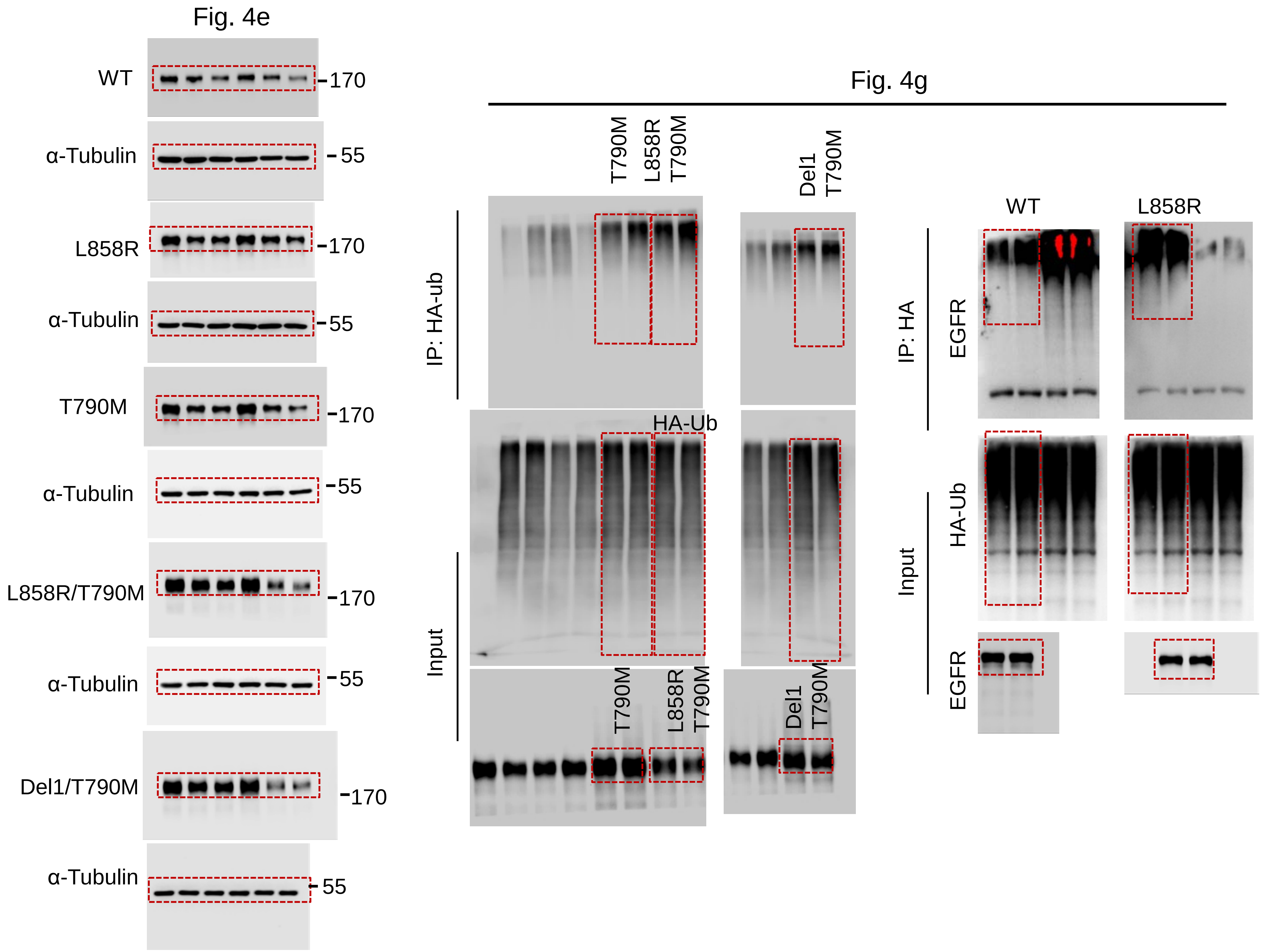

Fig. 4e
WT
170
α-Tubulin
55
L858R
170
α-Tubulin
55
T790M
170
α-Tubulin
55
L858R/T790M
170
α-Tubulin
55
Del1/T790M
170
α-Tubulin
55
Fig. 4g
T790M
L858R
T790M
Del1
T790M
IP: HA-ub
HA-Ub
Input
T790M
L858R
T790M
Del1
T790M
WT
L858R
IP: HA
EGFR
Input
HA-Ub
EGFR

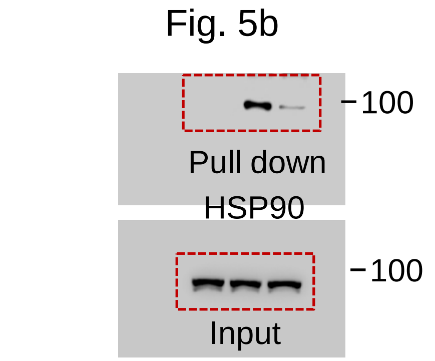
Fig. 5b
100
Pull down
HSP90
100
Input

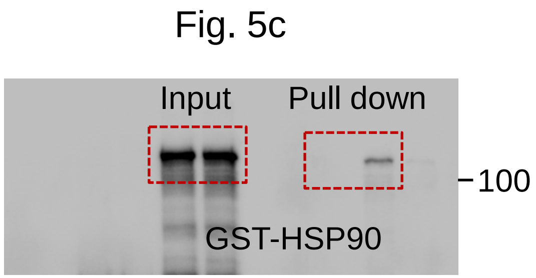
Fig. 5c
Input
Pull down
100
GST-HSP90

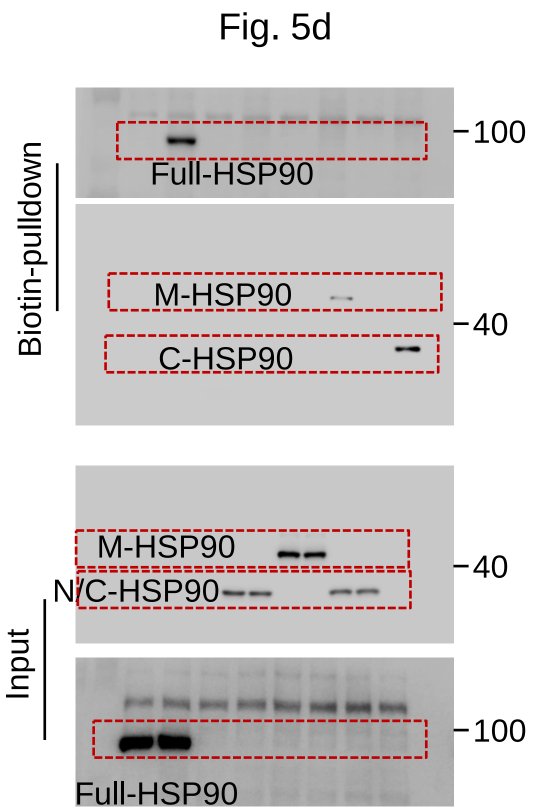
Fig. 5d
Biotin-pulldown
100
Full-HSP90
M-HSP90
40
C-HSP90
Input
M-HSP90
40
N/C-HSP90
100
Full-HSP90

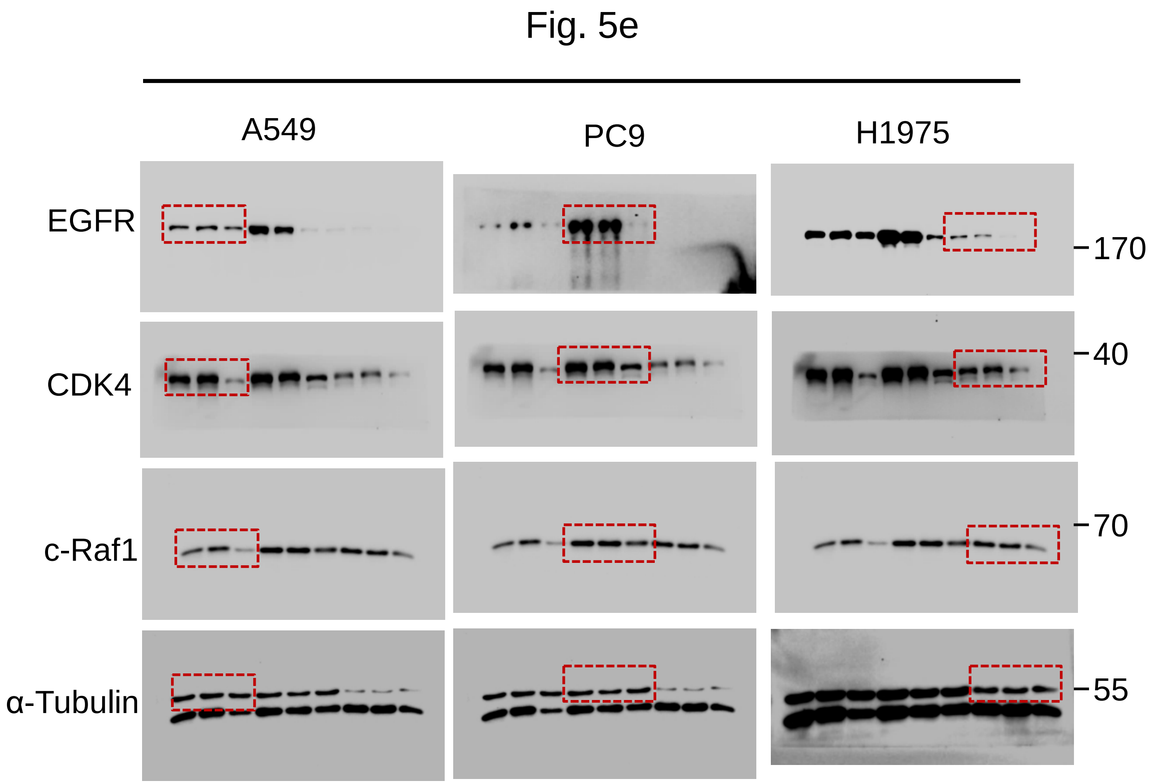
Fig. 5e
A549
PC9
H1975
EGFR
170
CDK4
40
c-Raf1
70
α-Tubulin
55

Fig. 5f

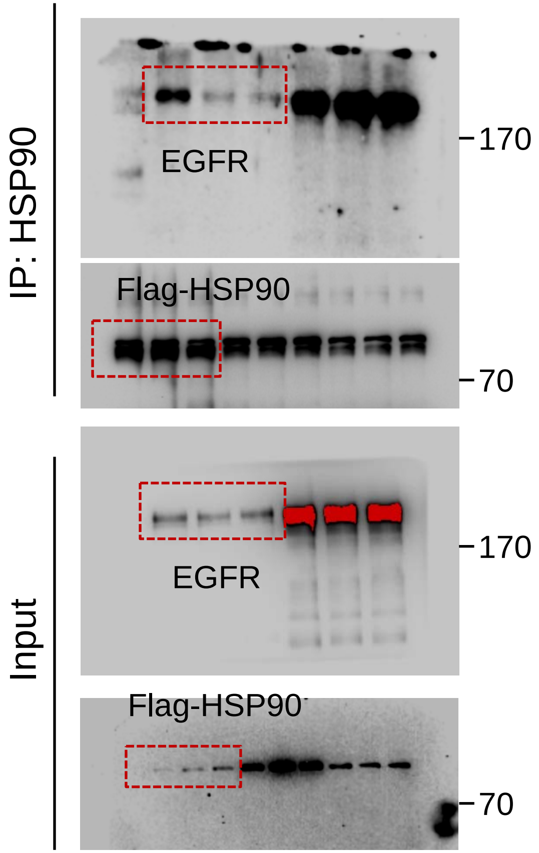
IP: HSP90
EGFR
170
Flag-HSP90
70
Input
EGFR
170
Flag-HSP90
70

Fig. 5g

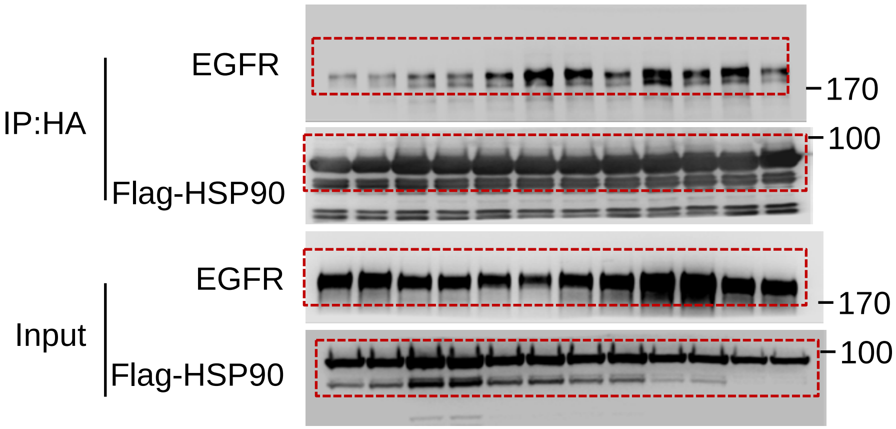
IP:HA
EGFR
170
Flag-HSP90
100
Input
EGFR
170
Flag-HSP90
100

Fig. 6e

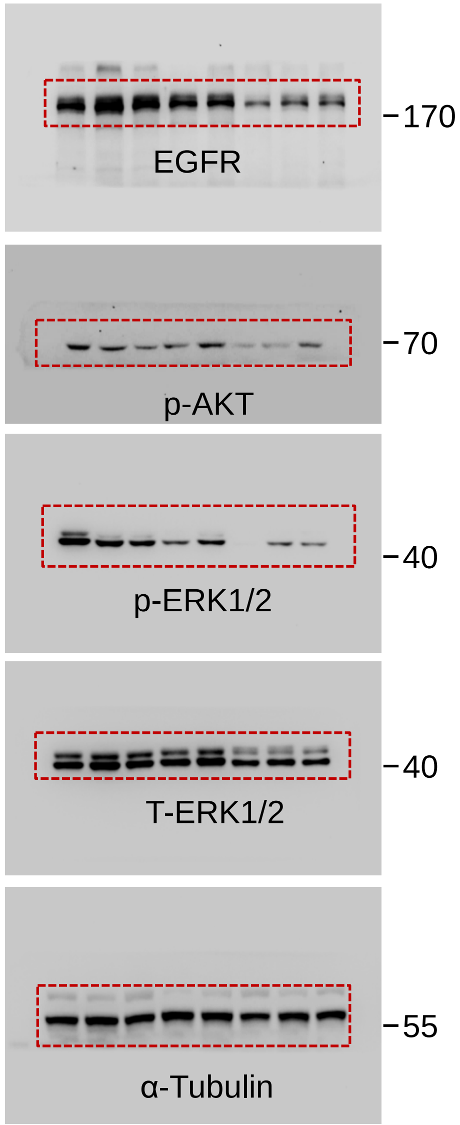

Fig. 7b

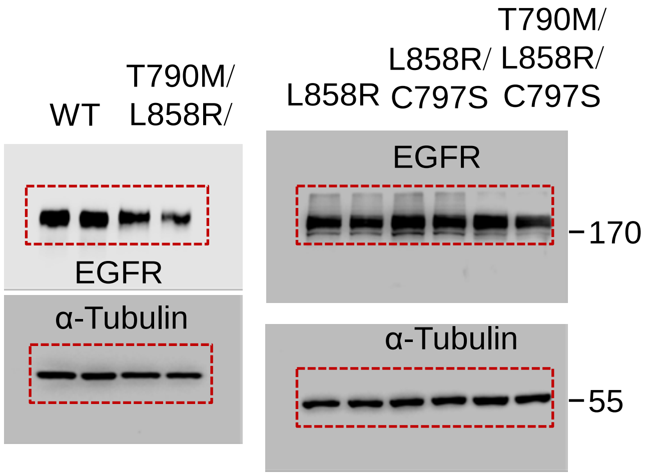

Fig. 7c

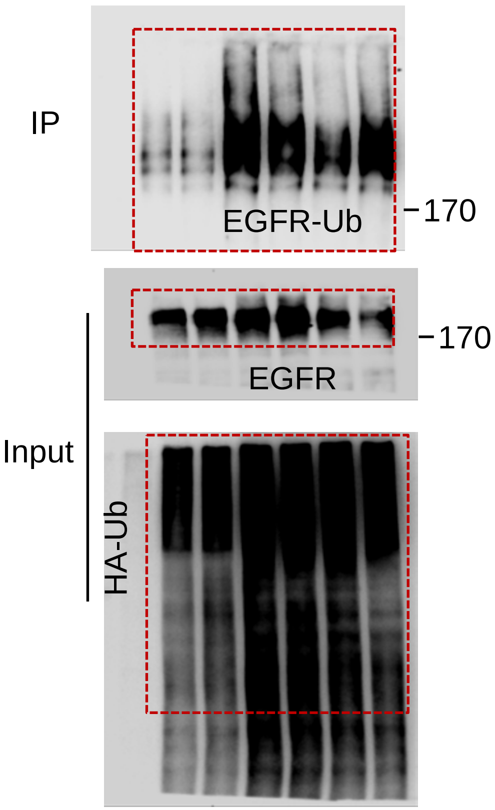

Supplementary Fig. 17a

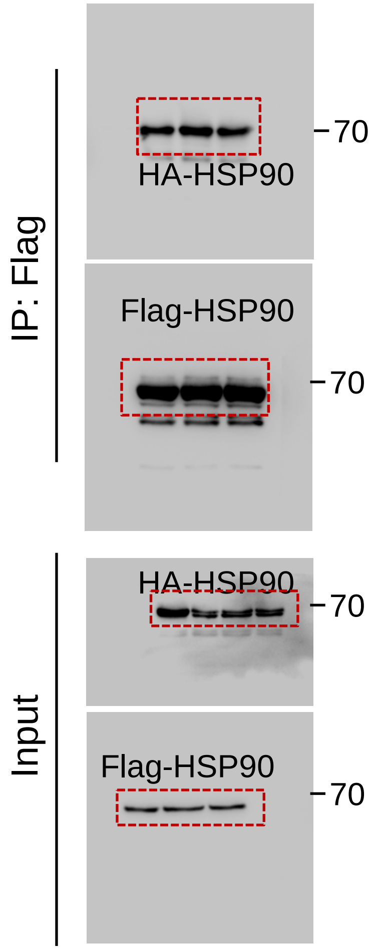

Supplementary Fig. 17b-c

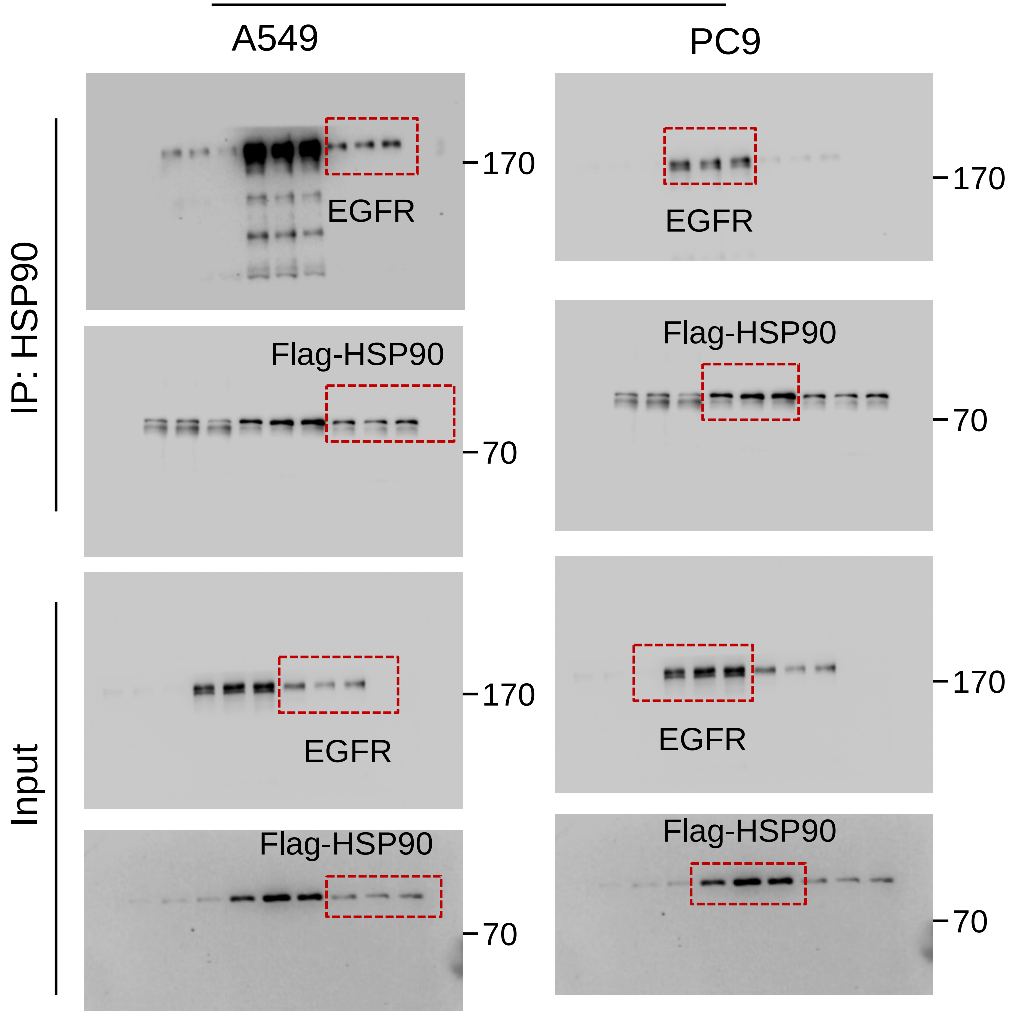

Supplementary table 1: Antibodies used in this study

Antibody	Source	Dilutions
(pSer473)-AKT	CST (#4060S)	WB (1:1,000)
(pThr308)-AKT	CST (#13038)	WB (1:1,000)
AKT	CST (#4691)	WB (1:1,000)
(pThr202/Tyr204) ERK1/2	CST(#4370S)	WB (1:1,000)
ERK1/2	CST (#4695S)	WB (1:1,000)
HA-tag	Sigma-Aldrich (H3663)	WB (1:5,000)
FLAG-tag	Sigma-Aldrich (F3165)	WB(1:5,000)
α-Tubulin	Beyotime (AT819)	WB (1:5,000)
GAPDH	Beyotime (AG019)	WB (1:5,000)
Anti-rabbit IgG	Jackson (11-035-003)	WB (1:10,000)
Anti-mouse IgG	Jackson (15-035-003)	WB (1:10,000)
CDK4	Bimake (A5189)	WB (1:1,000)
c-Raf	Bimake (A5043)	WB (1:1,000)
EGFR	Santa Cruz (sc-373746)	WB (1:3,000)
EGFR	Abcam (ab32077)	IF (1:100)
HSP90	Bimake (A5088)	WB (1:1,000)
pY527-Src	CST (#2105)	WB (1:1,000)
pY397-FAK	CST (#3283)	WB (1:1,000)

Abbreviations: WB (Western blotting); IP (immunoprecipitation);

IF (immunofluorescence).

Supplementary methods

Extraction, isolation and structure identification of natural D6-1

The roots of *Codonopsis pilosula* (16.0 kg) were powdered and extracted under reflux with 80% EtOH (3 × 40 L × 1 h) to give a crude extract, which was suspended in water followed by successive partition with petroleum ether and EtOAc to afford an EtOAc soluble extract (220 g). This extract was divided into nine parts (Frs. A–I) by a MCI gel CHP 20P column eluted with aqueous MeOH (5%–100%). Fr.E (10.0 g) was submitted to a RP-18 column eluted with gradient aqueous MeOH (10%–100%) to yield 4 subfractions (Fr.E.1-Fr.E.4). Fr.E.4 (2.2 g) was separated by Sephadex LH-20 (MeOH) followed by semi-preparative HPLC (CH_3CN/H_2O, 40:60) to give **D6-1** (2.0 mg, t_R = 20.2 min). **D6-1**. ESIMS *m/z* $[M + Na]^+$ 303; ^{1}H NMR (MeOD, 600 MHz) δ: 8.44 (d, *J* = 5.0 Hz, 1H, H-3), 8.33 (d, *J* = 5.0 Hz, 1H, H-4), 8.24 (brd, *J* = 7.8 Hz, 1H, H-5), 7.61 (brt, *J* = 7.8 Hz, 1H, H-7), 7.73 (brd, *J* = 7.8 Hz, 1H, H-8), 7.55 (d, *J* = 12.1 Hz, 1H, H-11), 7.33 (brt, *J* = 7.8 Hz, 1H, H-6), 6.48 (d, *J* = 12.1 Hz, 1H, H-12), 3.64 (s, 3H, H-14); ^{13}C NMR (MeOD, 150 MHz) δ: 195.4 (C-10), 168.3 (C-13), 143.6 (C-8a), 139.3 (C-3), 138.7 (C-11), 136.9 (C-9a), 136.6 (C-1), 133.4 (C-4a), 130.4 (C-7), 128.8 (C-12), 122.7 (C-5), 121.8 (C-6), 121.7 (C-5a), 120.4 (C-4), 113.6 (C-8), 52.4 (C-14). Detailed compound characterization data are presented in Supplementary Figures 1 to 6.

Preparation of D6

The target compound **D6** was accomplished by a four-step reaction with a 10% yield. The detailed reaction processes are as follows:

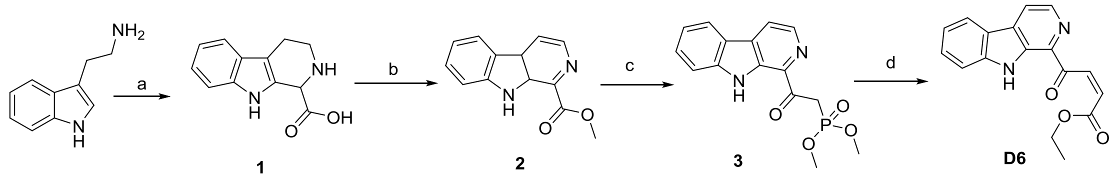

Scheme 1. Synthesis pathway of **D6**. Reagents and condition: (a) glyoxalic acid, EA, H_2O, K_2CO_3, RT, 24 h; (b) $SOCl_2$, MeOH, RT, 4 h, reflux 4 h; DBU, DMSO, RT, 48 h; (c) dimethyl methylphosphonate, THF, BuLi, −78 ℃, 1.5 h; (d) t-BuOK, ethyl acetalate, ℃, 1.5 h.

Synthesis of ethyl (*Z*)-4-oxo-4-(9*H*-pyrido[3,4-b]indol-1-yl)but-2-enoate (D6)

Glyoxalic acid (4.9 g, 70 mmol) dissolved in water (70 mL) was added to a solution of tryptamine (9.6 g, 60 mmol) in 145 mL of ethyl acetate (EA) with intense stirring. The pH was adjusted to 5.0 by adding 10% K_2CO_3 aqueous solution over 24 h. The filtrated solids were collected and washed in a small volume of EA before vacuum drying to afford compound **1** (9.08 g, 70% yield).

$SOCl_2$ (5 mL) at room temperature was dropped slowly into a solution of compound **1** (9.0 g, 42 mmol) in 200 mL of dry methanol and stirred for 4 h. Then a reflux reaction was conducted for 4 h before thin layer chromatography (TLC) to complete the reaction. The reaction mixture was concentrated under reduced pressure and dried before proceeding directly to the next reaction.

1,8-diazabicyclo[5.4.0]undec-7-ene (DBU) (7.0 g, 46 mmol) was added to a solution of the methyl ester of compound **1** (7.6 g, 33 mmol) in 50 mL of DMSO. After stirring for 48 h, the solution was poured into 400 mL ice-cold water and the reaction mixture was successively extracted with EA and dichloromethane (DCM). After drying with anhydrous sodium sulfate, the extraction solution was rotated and dried by silica gel column chromatography to obtain compound **2** (6.02 g, 80% yield).

BuLi (65 mL, 1.6 M, 105 mmol) at −78 ℃ was added to a solution of dimethyl methylphosphonate (13.0 g, 105 mmol) in 150 mL of dry tetrahydrofuran (THF), and stirred for 0.5 h. Then, compound **2** (6.0 g, 26.3 mmol) was dissolved in 50 mL dry THF and added to the above reaction solution, and stirred for 1 h at −78 ℃. The reaction system was added to 100 mL saturated ammonium chloride solution and extracted three times with EA. The extracted phase was combined, dried by anhydrous sodium sulfate, concentrated, and then residue silica gel column chromatography was performed to obtain compound **3** (4.18 g, 50% yield).

t-BuOK (1.7 g, 15 mmol) at 0 ℃ temperature was added to a solution of compound **3** (4.0 g, 12.5 mmol) in 100 mL of dry THF and stirred for 0.5 h. Then a solution of excessive EA in toluene was added to the above reaction solution, and stirred for 1 h at room temperature. The reaction system was added to 100 mL saturated ammonium chloride solution and extracted three times with EA. The extracted phase was

combined, dried by anhydrous sodium sulfate, concentrated, and residue silica gel column chromatography was performed to obtain **D6** (1.47 g, 10% yield).

ESIMS *m/z* $[M + H]^+$ 295; HRESIMS, calculated for $C_{17}H_{14}N_2O_3$: $[M + H]^+$ 295.1080; found: 295.1074; ^1H NMR ($CDCl_3$, 600 MHz) δ: 10.31 (brs, 1H, *N*H), 8.51 (d, *J* = 4.8 Hz, 1H, H-3), 8.11 (d, *J* = 4.8 Hz, 1H, H-4), 8.12 (brd, *J* = 7.8 Hz, 1H, H-5), 7.59 (brt, *J* = 7.8 Hz, 1H, H-7), 7.55 (brd, *J* = 7.8 Hz, 1H, H-8), 7.53 (d, *J* = 12.2 Hz, 1H, H-11), 7.32 (brt, *J* = 7.8 Hz, 1H, H-6), 6.44 (d, *J* = 12.2 Hz, 1H, H-12), 4.15 (q, *J* = 7.1 Hz, 2H, H-14), 1.16 (t, *J* = 7.1 Hz, 3H, H-15); ^{13}C NMR ($CDCl_3$, 150 MHz) δ: 195.1 (C-10), 166.1 (C-13), 141.3 (C-8a), 138.5 (C-3), 135.9 (C-9a), 137.6 (C-11), 135.8 (C-1), 131.8 (C-4a), 129.5 (C-7), 128.5 (C-12), 122.0 (C-5), 121.0 (C-6), 120.7 (C-5a), 119.4 (C-4), 112.2 (C-8), 61.1 (C-14), 14.0 (C-15).

Preparation of biotin labeled D6 (D6-4)

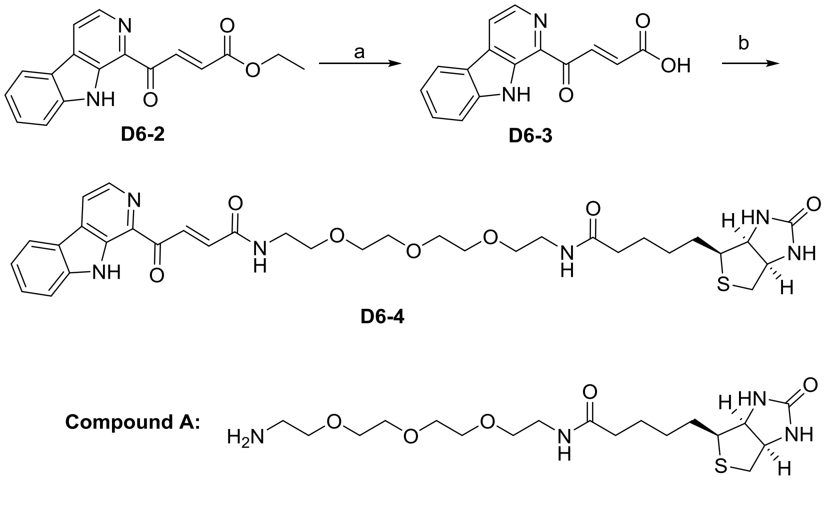

Scheme 2. Synthesis pathway of **D6-4**.
Reagents and condition: (a) K_2CO_3, THF, H_2O, RT, 1 h; (b) HATU, Et_3N, DMF, RT.

Synthesis of *N*-((*E*)-13,16-dioxo-16-(9*H*-pyrido[3,4-b]indol-1-yl)-3,6,9-trioxa-12-azahexadec-14-en-1-yl)-5-((3a*S*,4*S*,6a*R*)-2-oxohexahydro-1*H*-thieno[3,4-d]imidazol-4-yl)pentanamide (D6-4)

The *cis* form of **D6** is readily transformed to the *trans* form during the chemical synthesis of biotin-labeled **D6** and the actual form of **D6** during cell culture is *trans*

form (Supplementary Fig. 18–19). As such, preparation of biotin labeled **D6** started from the stable form of **D6** (**D6-2**). In detail, 2 mL H_2O, K_2CO_3 (27.6 mg) was added to a solution of **D6-2** (28 mg, 0.01 mmol) in 2 mL THF at ambient temperature and stirred until TLC showed complete reaction of raw material. Then, the THF was removed by vacuum distillation. Yellow solids precipitated upon addition of 1 mol/L HCl to acidize the solution. The precipitate was collected by centrifugation to afford 12.0 mg **D6-3**.

Without further purification, **D6-3** was used in the next reaction. To a solution of 12.0 mg **D6-3** in 5 mL dimethylformamide (DMF), 38 mg *O*-(7-azabenzotriazol-1-yl)-*N*,*N*,*N*,*N*-tetramethyl uronium hexafluorophosphate (HATU) was added at 0°C and stirred for 30 min. Then **compound A** (20 mg) and triethylamine (30 mg) were added to the mixture and stirred at room temperature for 6 h. The mixture was poured into 10 mL ice-cold water and extracted with 10 mL EA. The organic layer was washed twice with 5 mL saturated sodium chloride solution, dried over Na_2SO_4 and evaporated. The residue was purified by chromatography on a silica gel column using DCM and MeOH (v/v: 20/1) as the eluents to afford **D6-4** as a yellow powder (15.7 mg, 52% yield).

ESIMS *m/z* $[M + H]^+$ 667; HRESIMS, calcd for $C_{33}H_{42}N_6O_7S$: $[M + H]^+$ 667.2910; found: 667.2909; ^{1}H NMR (600 MHz, $CDCl_3$) δ 10.66 (s, 1H), 8.73 (d, J = 15.7 Hz, 1H), 8.57 (d, J = 4.8 Hz, 1H), 8.16 (dd, J = 12.6, 6.4 Hz, 3H), 7.69–7.65 (m, 2H), 7.61 (d, J = 7.5 Hz, 1H), 7.57–7.50 (m, 1H), 7.47 (td, J = 7.6, 2.9 Hz, 1H), 7.34 (t, J = 7.5 Hz, 1H), 7.23 (d, J = 15.7 Hz, 1H), 4.53–4.41 (m, 1H), 4.33–4.25 (m, 1H), 3.65–3.62 (m, 4H), 3.60 (t, J = 5.0 Hz, 4H), 3.48 (dd, J = 10.3, 5.2 Hz, 2H), 3.16–3.04 (m, 1H), 2.88 (dd, J = 12.9, 4.9 Hz, 1H), 2.70 (d, J = 12.8 Hz, 1H), 2.21 (t, J = 7.2 Hz, 2H), 2.04 (s, 2H), 1.46–1.36 (m, 2H); ^{13}C NMR ($CDCl_3$, 150 MHz) δ: 192.6, 173.4, 165.2, 152.0, 141.5, 138.5, 136.5, 135.8, 135.5, 132.4, 131.9, 129.6, 122.0, 121.1, 120.8, 119.7, 112.5, 70.6, 70.5, 70.2, 70.2, 70.1, 69.9, 61.9, 60.6, 60.3, 55.4, 40.7, 39.8, 39.3, 35.9, 28.1, 25.5. The structure of **D6-4** was confirmed by spectroscopic methods including ^{1}H (Supplementary Fig. 15), ^{13}C NMR (Supplementary Fig. 16), and HRESIMS spectrum (Supplementary Fig. 17).
