# Supplementary material for: A small-molecule compound D6 overcomes EGFR-T790M-mediated resistance in non-small cell lung cancer
Source: Commun Biol. 2021 Dec 13;4:1391. doi: 10.1038/s42003-021-02906-4 (PMC8668973; doi:10.1038/s42003-021-02906-4)
Supplement: Supplementary file 4 — Reporting summary. [file 42003_2021_2906_MOESM4_ESM.pdf]

Corresponding author(s): Baohua Liu

Last updated by author(s): Sept 27, 2021

Reporting Summary

Nature Portfolio wishes to improve the reproducibility of the work that we publish. This form provides structure for consistency and transparency in reporting. For further information on Nature Portfolio policies, see our Editorial Policies and the Editorial Policy Checklist.

Statistics

For all statistical analyses, confirm that the following items are present in the figure legend, table legend, main text, or Methods section.

n/a	Confirmed	
☐	☒	The exact sample size (*n*) for each experimental group/condition, given as a discrete number and unit of measurement
☐	☒	A statement on whether measurements were taken from distinct samples or whether the same sample was measured repeatedly
☐	☒	The statistical test(s) used AND whether they are one- or two-sided<br>*Only common tests should be described solely by name; describe more complex techniques in the Methods section.*
☐	☒	A description of all covariates tested
☐	☒	A description of any assumptions or corrections, such as tests of normality and adjustment for multiple comparisons
☐	☒	A full description of the statistical parameters including central tendency (e.g. means) or other basic estimates (e.g. regression coefficient) AND variation (e.g. standard deviation) or associated estimates of uncertainty (e.g. confidence intervals)
☐	☒	For null hypothesis testing, the test statistic (e.g. *F*, *t*, *r*) with confidence intervals, effect sizes, degrees of freedom and *P* value noted<br>*Give P values as exact values whenever suitable.*
☒	☐	For Bayesian analysis, information on the choice of priors and Markov chain Monte Carlo settings
☒	☐	For hierarchical and complex designs, identification of the appropriate level for tests and full reporting of outcomes
☒	☐	Estimates of effect sizes (e.g. Cohen's *d*, Pearson's *r*), indicating how they were calculated

Our web collection on statistics for biologists contains articles on many of the points above.

Software and code

Policy information about availability of computer code

Data collection	Immunoblots data were collected by ChemiDoc Touch Imaging System (BIO-RAD).<br>IHC images were taken by BX43 (OLYMPUS).<br>Real-time PCR data were collected by the BIO-RAD CFX Connect (BIO-RAD).<br>Cell viability detection was performed by the EnSpire (Perkin Elmer Life Science).
Data analysis	Western blots are analyzed by Image Lab Software V5.2.1 build 11(BIO-RAD).<br>Image J soft is used for quantification analysis.<br>Microsoft Excel is applied for statistical analysis.

For manuscripts utilizing custom algorithms or software that are central to the research but not yet described in published literature, software must be made available to editors and reviewers. We strongly encourage code deposition in a community repository (e.g. GitHub). See the Nature Portfolio guidelines for submitting code & software for further information.

Data

Policy information about availability of data

All manuscripts must include a data availability statement. This statement should provide the following information, where applicable:

- Accession codes, unique identifiers, or web links for publicly available datasets
- A description of any restrictions on data availability
- For clinical datasets or third party data, please ensure that the statement adheres to our policy

Data supporting this study are available in the manuscript, supplementary information or provided within the source data file.

Field-specific reporting

Please select the one below that is the best fit for your research. If you are not sure, read the appropriate sections before making your selection.

- [x] Life sciences
- [ ] Behavioural & social sciences
- [ ] Ecological, evolutionary & environmental sciences

For a reference copy of the document with all sections, see nature.com/documents/nr-reporting-summary-flat.pdf

Life sciences study design

All studies must disclose on these points even when the disclosure is negative.

Sample size	Sample size was not predetermined by statistical methods. We chose the proper sample size (n >= 3 for most in vitro experiments) based on general rules or following the literature in this filed.
Data exclusions	No data are excluded.
Replication	Experiments were successfully performed for at least 3 replications.
Randomization	For cellular experiments, cells were randomly plated. For animal experiments, mice were randomized into different experimental groups.
Blinding	The data collection and analysis were not performed blind due to obvious differences between groups.

Reporting for specific materials, systems and methods

We require information from authors about some types of materials, experimental systems and methods used in many studies. Here, indicate whether each material, system or method listed is relevant to your study. If you are not sure if a list item applies to your research, read the appropriate section before selecting a response.

Materials & experimental systems

n/a	Involved in the study	
	x	Antibodies
	x	Eukaryotic cell lines
x		Palaeontology and archaeology
	x	Animals and other organisms
x		Human research participants
x		Clinical data
x		Dual use research of concern

Methods

n/a	Involved in the study	
x		ChIP-seq
x		Flow cytometry
x		MRI-based neuroimaging

Antibodies

Antibodies used

(pSer9)-GSK3β, CST (#9322), WB (1:1,000);
GSK3β, CST (#12456), WB (1:1,000);
(pSer473)-AKT, CST (#4060S), WB (1:1,000);
(pThr308)-AKT , CST (#13038), WB (1:1,000);
AKT, CST (#4691), WB (1:1,000);
(pThr202/Tyr204) ERK1/2, CST(#4370S) , WB (1:1,000);
ERK1/2, CST (#4695S), WB (1:1,000);
HA-tag, Sigma–Aldrich (H3663), WB (1:5,000);
FLAG-tag, Sigma–Aldrich (F3165), WB(1:5,000);
α-Tubulin, Beyotime (AT819) , WB (1:5,000);
GAPDH, Beyotime (AG019), WB (1:5,000);
HSP90, Bimake (A5088), WB (1:1000);
EGFR, Santa Cruz (sc-373746), WB(1:3000);
EGFR, Abcam (ab32077), WB (1:5000), IF (1:100);
cleaved PARP, CST (#9548), WB (1:1000);
c-Raf, Bimake (A5043), WB (1:1000);
CDK4, Bimake (A5189), WB (1:1000);
Anti-mouse IgG, Jackson (11-035-003), WB(1:10,000);
Anti-rabbit IgG, Jackson (15-035-003), WB(1:10,000);
pY397-FAK, CST (#3283),WB (1:1000).

Validation

(pSer9)-GSK3β, CST (#9322), WB (1:1,000),

https://www.cellsignal.cn/products/primary-antibodies/phospho-gsk-3-beta-ser9-d3a4-rabbit-mab/9322

GSK3β, CST (#12456), WB (1:1,000);
https://www.cellsignal.cn/products/primary-antibodies/gsk-3b-d5c5z-xp-rabbit-mab/12456?site-search-type=Products&N=4294956287&Ntt=12456&fromPage=plp&_requestid=539453;

(pSer473)-AKT, CST (#4060S), WB (1:1,000),
https://www.cellsignal.cn/products/primary-antibodies/phospho-akt-ser473-d9e-xp-rabbit-mab/4060?site-search-type=Products&N=4294956287&Ntt=4060&fromPage=plp&_requestid=539501

(pThr308)-AKT , CST (#13038), WB (1:1,000),
https://www.cellsignal.cn/products/primary-antibodies/phospho-akt-thr308-d25e6-xp-rabbit-mab/13038?site-search-type=Products&N=4294956287&Ntt=13038&fromPage=plp&_requestid=539589

AKT, CST (#4691), WB (1:1,000),
https://www.cellsignal.cn/products/primary-antibodies/akt-pan-c67e7-rabbit-mab/4691?site-search-type=Products&N=4294956287&Ntt=4691&fromPage=plp&_requestid=539611

(pThr202/Tyr204) ERK1/2, CST(#4370S) , WB (1:1,000),
https://www.cellsignal.cn/products/primary-antibodies/phospho-p44-42-mapk-erk1-2-thr202-tyr204-d13-14-4e-xp-rabbit-mab/4370?site-search-type=Products&N=4294956287&Ntt=4370&fromPage=plp

ERK1/2, CST (#4695S), WB (1:1,000),
https://www.cellsignal.cn/products/primary-antibodies/p44-42-mapk-erk1-2-137f5-rabbit-mab/4695?site-search-type=Products&N=4294956287&Ntt=4695&fromPage=plp

HA-tag, Sigma–Aldrich (H3663), WB (1:5,000),
https://www.sigmaaldrich.cn/CN/en/product/sigma/h3663?context=product

FLAG-tag, Sigma–Aldrich (F3165), WB(1:5,000),
https://www.sigmaaldrich.cn/CN/en/product/sigma/f3165?context=product

α-Tubulin, Beyotime (AT819) , WB (1:5,000),
https://www.beyotime.com/product/AT819.htm

GAPDH, Beyotime (AG019), WB (1:5,000),
https://www.beyotime.com/product/AG019.htm

HSP90, Bimake (A5088), WB (1:1000),
https://www.bimake.cn/antibody/hsp90-beta-rabbit-recombinant-mab.html

EGFR, Santa Cruz (sc-373746), WB(1:3000),
https://www.scbt.com/p/egfr-antibody-a-10

EGFR, Abcam (ab32077), WB (1:5000), IF (1:100),
https://www.abcam.com/egfr-antibody-e235-ab32077.html

cleaved PARP, CST (#9548), WB (1:1000),
https://www.cellsignal.cn/products/primary-antibodies/cleaved-parp-asp214-7c9-mouse-mab-mouse-specific/9548?site-search-type=Products&N=4294956287&Ntt=9548&fromPage=plp&_requestid=540213

c-Raf, Bimake (A5043), WB (1:1000),
https://www.bimake.cn/antibody/raf1-rabbit-recombinant-mab.html

CDK4, Bimake (A5189), WB (1:1000),
https://www.bimake.cn/antibody/cdk4-rabbit-recombinant-mab.html

Anti-rabbit IgG, Jackson (11-035-003), WB(1:10,000),
https://www.jacksonimmuno.com/catalog/products/111-035-003

Anti-mouse IgG, Jackson (15-035-003), WB(1:10,000),
https://www.jacksonimmuno.com/catalog/products/115-035-003

pY397-FAK, CST (#3283),WB (1:1000),
https://www.cellsignal.cn/products/primary-antibodies/phospho-fak-tyr397-antibody/3283?site-search-type=Products&N=4294956287&Ntt=3283&fromPage=plp&_requestid=540569.

Eukaryotic cell lines

Policy information about cell lines

Cell line source(s)	HEK293, A549, LO2 and PC9 cells were obtained from American Type Culture Collection. MRC5 and NCI H1975 cells were purchased from Chinese Academy of Sciences Committee Type Culture Collection Cell Bank.
Authentication	All cell lines were routinely authenticated by analysis of cell growth rate and morphology.
Mycoplasma contamination	All cell lines were routinely tested to ensure the free of mycoplasma contamination.
Commonly misidentified lines (See ICLAC register)	None.

Animals and other organisms

Policy information about studies involving animals; ARRIVE guidelines recommended for reporting animal research

Laboratory animals	Athymic nu/nu mice, BALB/cJ, 6-8 weeks, males.
Wild animals	No wild animals were used in this study.
Field-collected samples	This study did not involve any field-collected samples.
Ethics oversight	All the experimental animals were housed and handled in accordance with protocols approved by the Committee on the Use of Live Animals in Teaching and Research of Shenzhen University.

Note that full information on the approval of the study protocol must also be provided in the manuscript.
